# PolyGR and polyPR knock-in mice reveal a conserved neuroprotective extracellular matrix signature in *C9orf72* ALS/FTD neurons

**DOI:** 10.1038/s41593-024-01589-4

**Published:** 2024-02-29

**Authors:** Carmelo Milioto, Mireia Carcolé, Ashling Giblin, Rachel Coneys, Olivia Attrebi, Mhoriam Ahmed, Samuel S. Harris, Byung Il Lee, Mengke Yang, Robert A. Ellingford, Raja S. Nirujogi, Daniel Biggs, Sally Salomonsson, Matteo Zanovello, Paula de Oliveira, Eszter Katona, Idoia Glaria, Alla Mikheenko, Bethany Geary, Evan Udine, Deniz Vaizoglu, Sharifah Anoar, Khrisha Jotangiya, Gerard Crowley, Demelza M. Smeeth, Mirjam L. Adams, Teresa Niccoli, Rosa Rademakers, Marka van Blitterswijk, Anny Devoy, Soyon Hong, Linda Partridge, Alyssa N. Coyne, Pietro Fratta, Dario R. Alessi, Ben Davies, Marc Aurel Busche, Linda Greensmith, Elizabeth M. C. Fisher, Adrian M. Isaacs

**Affiliations:** 1 UK Dementia Research Institute, University College London, London, UK; 2 Department of Neurodegenerative Disease, UCL Queen Square Institute of Neurology, London, UK; 3 UCL Institute of Healthy Ageing, Department of Genetics, Evolution and Environment, University College London, London, UK; 4 Department of Neuromuscular Diseases, UCL Queen Square Institute of Neurology, London, UK; 5 Aligning Science Across Parkinson’s (ASAP) Collaborative Research Network, Chevy Chase, MD USA; 6 Medical Research Council (MRC) Protein Phosphorylation and Ubiquitylation Unit, School of Life Sciences, University of Dundee, Dundee, UK; 7 Wellcome Centre for Human Genetics, University of Oxford, Oxford, UK; 8 Research Support Service, Institute of Agrobiotechnology, CSIC-Government of Navarra, Mutilva, Spain; 9 Department of Neuroscience, Mayo Clinic, Jacksonville, FL USA; 10 VIB Center for Molecular Neurology, University of Antwerp, Antwerp, Belgium; 11 Department of Biomedical Sciences, University of Antwerp, Antwerp, Belgium; 12 UK Dementia Research Institute, Maurice Wohl Clinical Neuroscience Institute, Institute of Psychiatry, Psychology and Neuroscience, King’s College London, London, UK; 13 Department of Neurology, Johns Hopkins University School of Medicine, Baltimore, MD USA; 14 Brain Science Institute, Johns Hopkins University School of Medicine, Baltimore, MD USA; 15 UCL Queen Square Motor Neuron Disease Centre, UCL Queen Square Institute of Neurology, London, UK; 16 Francis Crick Institute, London, UK

**Keywords:** Amyotrophic lateral sclerosis, Neurodegeneration

## Abstract

Dipeptide repeat proteins are a major pathogenic feature of *C9orf72* amyotrophic lateral sclerosis (C9ALS)/frontotemporal dementia (FTD) pathology, but their physiological impact has yet to be fully determined. Here we generated *C9orf72* dipeptide repeat knock-in mouse models characterized by expression of 400 codon-optimized polyGR or polyPR repeats, and heterozygous *C9orf72* reduction. (GR)400 and (PR)400 knock-in mice recapitulate key features of C9ALS/FTD, including cortical neuronal hyperexcitability, age-dependent spinal motor neuron loss and progressive motor dysfunction. Quantitative proteomics revealed an increase in extracellular matrix (ECM) proteins in (GR)400 and (PR)400 spinal cord, with the collagen COL6A1 the most increased protein. TGF-β1 was one of the top predicted regulators of this ECM signature and polyGR expression in human induced pluripotent stem cell neurons was sufficient to induce TGF-β1 followed by COL6A1. Knockdown of TGF-β1 or COL6A1 orthologues in polyGR model *Drosophila* exacerbated neurodegeneration, while expression of TGF-β1 or COL6A1 in induced pluripotent stem cell-derived motor neurons of patients with C9ALS/FTD protected against glutamate-induced cell death. Altogether, our findings reveal a neuroprotective and conserved ECM signature in C9ALS/FTD.

## Abstract (web summary)

*C9orf72* ALS/FTD polyGR and polyPR knock-in mice show cortical hyperexcitability and motor neuron loss accompanied by an increase in extracellular matrix proteins in the spinal cord that is conserved in patient iPS cell-derived neurons and is neuroprotective.

## Main

Amyotrophic lateral sclerosis (ALS), characterized by progressive muscle weakness and atrophy, and FTD, characterized by behavioral change or language dysfunction, share overlapping clinical, pathological and genetic features. A GGGGCC (G_4_C_2_) repeat expansion in the first intron of the *C9orf72* gene is the most common genetic cause of ALS and FTD^1,2^, collectively termed C9ALS/FTD. Three mechanisms have been proposed to induce C9ALS/FTD pathology: (1) reduced transcription of *C9orf72*; (2) the presence of sense and antisense repeat-containing RNA; and (3) expression of aberrant dipeptide repeat (DPR) proteins encoded in six frames by the hexanucleotide repeat^3^.

The DPRs are derived from sense and antisense repeat-containing RNAs, which are translated by repeat-associated non-ATG initiated (RAN) translation, a non-canonical protein translation mechanism that does not require an ATG start codon^4^. RAN translation occurs in every reading frame, encoding five potentially toxic DPRs: polyGA, polyGR, polyGP, polyPA and polyPR, which all form neuronal cytoplasmic inclusions in the brains of patients with C9ALS/FTD (refs. ^5,6^). We and others have shown that DPR proteins are toxic in vivo and in vitro^3^. The arginine-rich DPR proteins, polyGR and polyPR, are generally the most toxic species in several systems, including *Drosophila*^7^, mammalian cells^8,9^ and mouse models^10–15^.

Several mouse models have been developed to elucidate pathomechanisms associated with C9ALS/FTD pathology^16,17^. *C9orf72* homozygous knockout mouse models manifest severe autoimmunity and lymphatic defects, indicating a role for C9orf72 in immune cell function, but there are no striking alterations in heterozygous knockouts which more closely resemble the level of C9orf72 depletion in patients^18,19^. Mouse models of *C9orf72* hexanucleotide repeat expansions have also been generated, taking advantage of either adeno-associated virus-mediated delivery^20,21^ or bacterial artificial chromosome (BAC) integration^22–25^. Although most of these mouse models recapitulate features of C9ALS/FTD, there is considerable phenotypic variation between them, possibly from site of integration, which may cause local mutation, copy number or other effects^26^. Finally, to better elucidate the role of DPRs in C9ALS/FTD, both viral and transgenic mouse models have been developed overexpressing codon-optimized constructs to synthesize only one specific DPR. Studies of these mice show that in vivo expression of polyGR throughout the mouse brain is toxic and results in several aspects of C9ALS/FTD pathology, including age-dependent neuronal loss, presence of cytoplasmic aggregates, development of anxiety-like behavior and social interaction defects^10–12^. Expression of polyPR is also toxic in mice. Similar to polyGR, polyPR mice have survival, motor and cognitive defects; hyperactivity and anxiety-like behavior; progressive brain atrophy; and neuronal loss^13–15^. Current in vivo and in vitro studies have identified common downstream molecular pathways that are dysregulated in C9ALS/FTD, including autophagy, nucleocytoplasmic transport, pre-messenger RNA splicing, stress granule dynamics, DNA damage repair, mitochondrial dysfunction, nuclear pore alterations and synaptic dysfunction^3,27,28^. However, the mechanism(s) by which the repeat expansion causes C9ALS/FTD when DPRs are expressed at more physiological levels are less clear.

The collection of mouse models produced to date have identified several potential pathomechanisms underlying C9ALS/FTD. However, the most relevant effects of DPRs in the endogenous context are not known. Thus, there is a need for refined models to understand the role of individual DPRs in vivo. Here, we generated *C9orf72* DPR knock-in mouse models characterized by expression of either polyGR or polyPR combined with heterozygous *C9orf72* reduction, to more accurately model DPR-induced dysfunction in C9ALS/FTD.

## Results

### Generation of *C9orf72* polyGR and polyPR knock-in mice

**Fig. 1:**
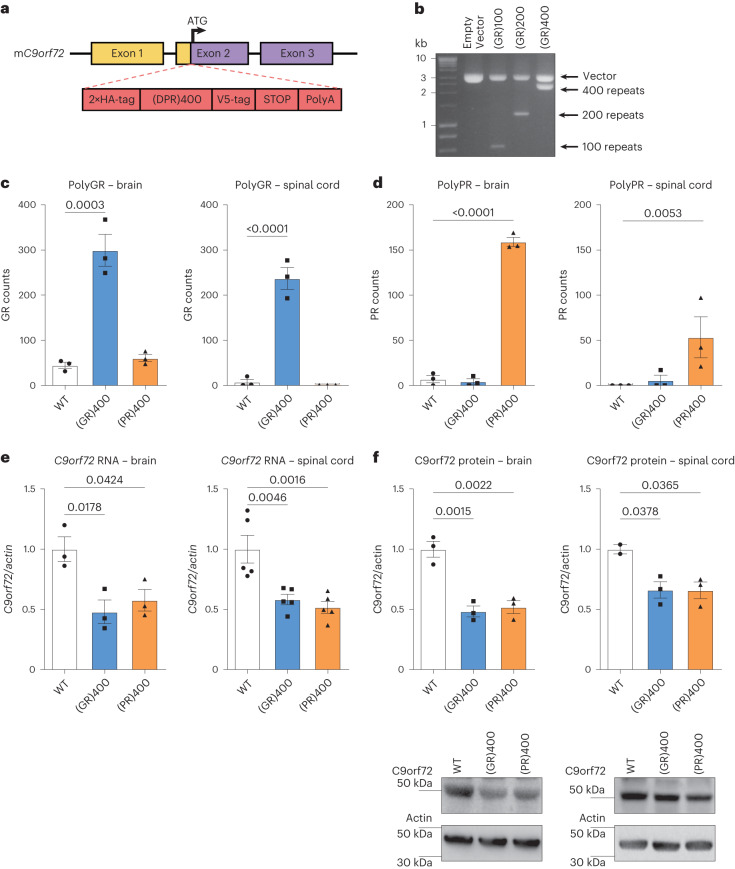
Generation of *C9orf72* polyGR and polyPR knock-in mice. **a**, Targeting strategy to generate (GR)400 and (PR)400 mice with the knock-in sequence inserted in exon 2 of mouse *C9orf72* immediately after, and in frame with, the endogenous ATG. Schematic shows the genomic region and the knock-in targeting construct. Exons are shown boxed; untranslated regions of exons are colored yellow with translated regions in purple. The targeting construct (in red) contains the knock-in sequence composed of a double HA-tag, 400 codon-optimized DPRs, a V5-tag, a stop codon and a 120-bp polyA tail. **b**, Agarose gel shows generation of patient-length polyGR, using recursive directional ligation to sequentially double the repeat length up to 400 repeats. Representative of at least *n* = 3 clones for each round of cloning. **c**, Quantification of polyGR proteins in brain (left panel) and spinal cord (right panel) of WT, (GR)400 and (PR)400 mice at 3 months of age by MSD immunoassay. Graph, mean ± s.e.m., *n* = 3 mice per genotype, one-way ANOVA, Bonferroni’s multiple comparison. **d**, Quantification of polyPR proteins in brain (left panel) and spinal cord (right panel) of WT, (GR)400 and (PR)400 mice at 3 months of age by MSD immunoassay. Graph, mean ± s.e.m., *n* = 3 mice per genotype, one-way ANOVA, Bonferroni’s multiple comparison. **e**, qPCR analysis of *C9orf72* transcript levels normalized to *β-actin* in brain (left panel) and spinal cord (right panel) of WT, (GR)400 and (PR)400 mice at 3 months of age. Graph, mean ± s.e.m.; *n* = 3 (brain), 5 (spinal cord) mice per genotype; one-way ANOVA, Bonferroni’s multiple comparison. **f**, Western blotting analysis of C9orf72 protein levels in brain (left panel) and spinal cord (right panel) of WT, (GR)400 and (PR)400 mice at 3 months of age. β-actin is shown as loading control. Graph, mean ± s.e.m.; *n* = 3 mice per genotype (left panel), *n* = 2 WT, 3 (GR)400 and 3 (PR)400 (right panel); one-way ANOVA, Bonferroni’s multiple comparison. Source data

**Extended Data Fig. 1:**
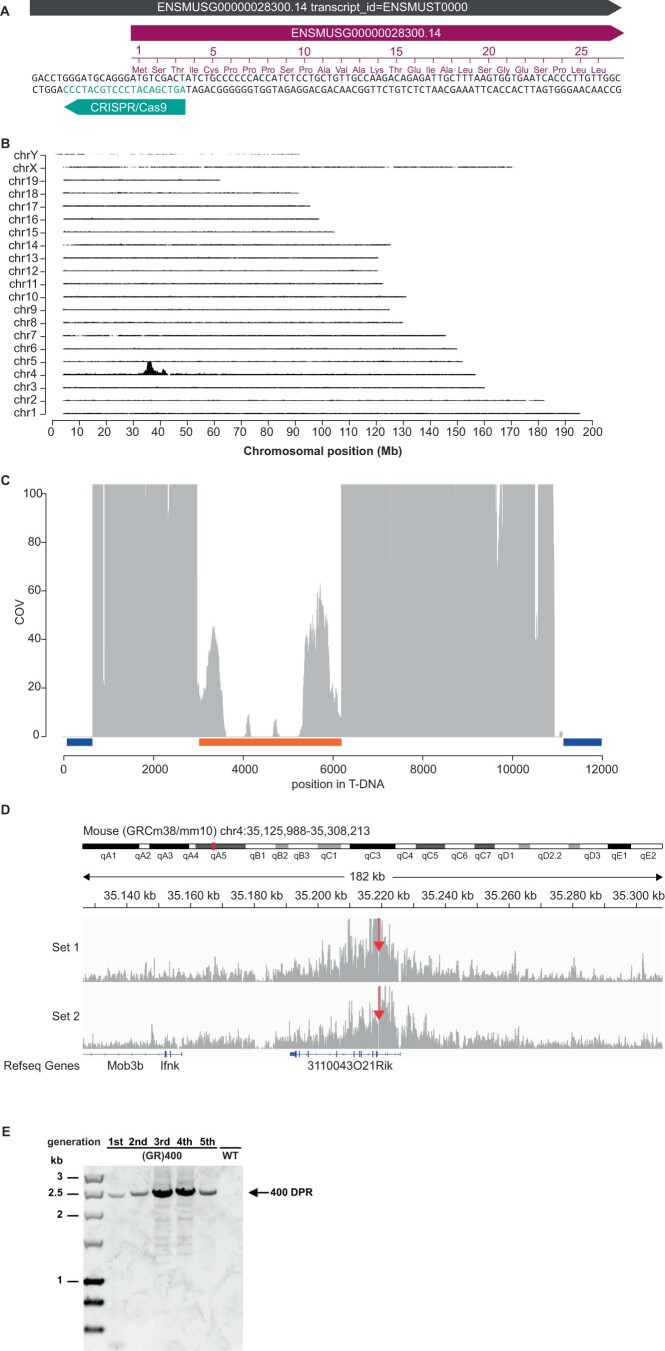
*C9orf72* knock-in strategy and confirmation. (**a**) Design for CRISPR assisted *C9orf72* gene targeting. The sgRNA for CRISPR/Cas9 is indicated by the teal bar. (**b**) Mapping of targeted locus amplification reads across the mouse genome. The chromosomes are indicated on the y-axis, the chromosomal position on the x-axis. (**c**) Targeted locus amplification sequence coverage across the knock-in sequence. The whole knock-in sequence has good coverage except for the blue underlined backbone sequences as expected from a correct targeting event as the backbone is not included. A coverage gap is present at the location of the 400 DPRs, and is underlined by the orange bar. Y-axis is limited to 100x. (**d**) Targeted locus amplification sequence coverage across the knock-in integration locus. The red arrow points toward the knock-in integration site. Y-axis is limited to 250x. (**e**) Repeat-length PCR of (GR)400 genomic DNA to establish repeat length and stability over five generations. All (GR)400 and (PR)400 mice in the study generated were genotyped for repeat length and showed the correct size.

**Extended Data Fig. 2:**
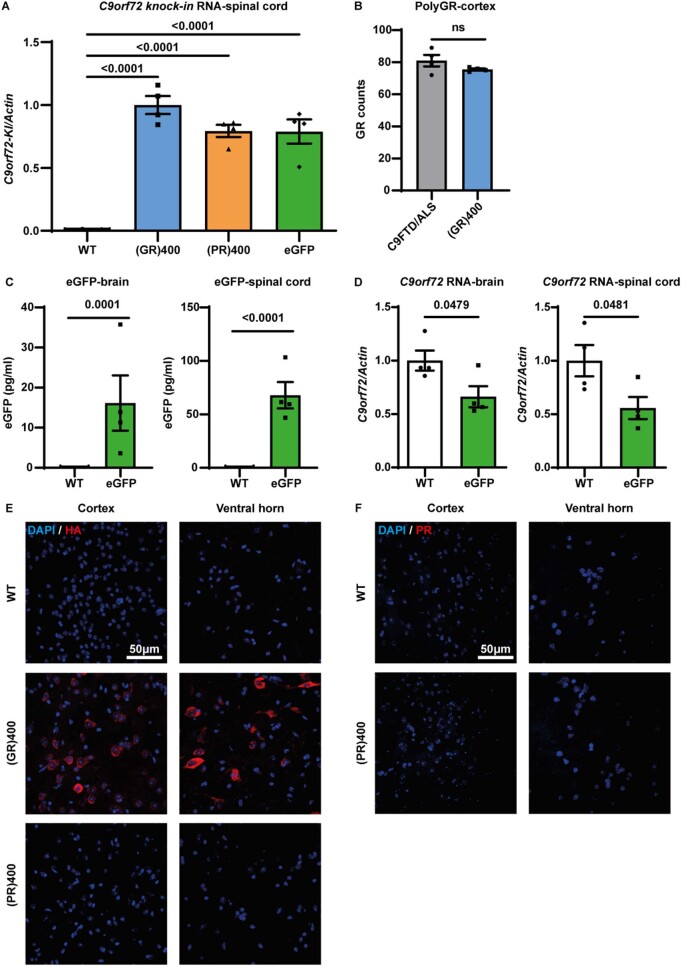
Patient and knock-in cortex polyGR comparison, generation of control *C9orf72*-eGFP knock-in mice, and *C9orf72* knock-in transgene transcript levels in spinal cord. (**a**) qPCR analysis of *C9orf72* knock-in transgene transcript levels normalised to *β-actin* in spinal cord of WT, (GR)400, (PR)400, and eGFP mice at 12 months of age. Common primers in the HA-tag and upstream endogenous mouse sequence were used to enable direct comparison between all lines. Graph, mean ± SEM, n = 4 mice per genotype, one-way ANOVA, Bonferroni’s multiple comparison. (**b**) Quantification of polyGR proteins in C9ALS/FTD patient and 12-month old (GR)400 mouse cortex by MSD immunoassay. Graph, mean ± SEM, n = 4 independent samples per experimental group, two-sided unpaired two-sample Student’s t-test, ns denotes p > 0.05. (**c**) Quantification of eGFP proteins in brain (left panel) and spinal cord (right panel) of WT and eGFP mice at 3 months of age by ELISA. Graph, mean ± SEM, n = 4 mice per genotype, two-sided unpaired two-sample Student’s t-test. (**d)** qPCR analysis of *C9orf72* transcript levels normalised to *β-actin* in brain (left panel) and spinal cord (right panel) of WT and eGFP mice at 3 months of age. Graph, mean ± SEM, n = 4 mice per genotype, two-sided unpaired two-sample Student’s t-test. (**e**) Representative confocal images of immunofluorescence staining labelled for HA-tag (red) in brain cortex and lumbar spinal cord ventral horn in WT (top panels), (GR)400 (centre panels), and (PR)400 (bottom panels) mice at 6 months of age. DAPI (blue) stains nuclei. n = 5 mice per genotype. (**f**) Representative confocal images of immunofluorescence staining labelled for PR (red) cellular localisation in brain cortex and spinal cord in WT (top panels) and (PR)400 (bottom panels) mice at 6 months of age. DAPI (blue) stains nuclei. n = 3 mice per genotype. Source data

As polyGR and polyPR are consistently damaging across model systems, we focused on generating polyGR and polyPR knock-in mice (Fig. 1a). We generated patient-length DPRs by performing recursive directional ligation^7,29^ to build 400 uninterrupted codon-optimized polyGR or polyPR repeats (Fig. 1b) flanked by epitope tags. We then used CRISPR–Cas9 to insert these repeats, or a control eGFP sequence, immediately after, and in frame with, the endogenous mouse *C9orf72* ATG start codon, in mouse embryonic stem cells (Extended Data Fig. 1a). We performed targeted locus amplification to identify clones with a single insertion site, and correct targeting, which maintained the integrity of both the knock-in sequences and the adjacent mouse genome (Extended Data Fig. 1b,d). Validated embryonic stem cell clones were used to generate knock-in mice using standard procedures. PCR amplification across the 400 codon-optimized repeats showed that DPR length is stable across at least five generations (Extended Data Fig. 1e). Quantitative PCR (qPCR) with reverse transcription showed comparable levels of (GR)400, (PR)400 and eGFP transcripts in the spinal cord (Extended Data Fig. 2a). As expected, (GR)400 and (PR)400 mice selectively expressed their own DPR in brain and spinal cord at 3 months of age (Fig. 1c,d). Importantly, levels of polyGR in (GR)400 mouse cortex were similar to C9ALS/FTD patient cortex (Extended Data Fig. 2b), confirming expression in the physiological range. (GR)400 and (PR)400 mice exhibited a significant ~40% reduction of *C9orf72* at messenger RNA and protein levels in 3-month-old brain and spinal cord (Fig. 1e,f), as predicted by our knock-in strategy, which inserts the DPR sequence into one copy of *C9orf72*, removing expression of *C9orf72* from that allele. Additionally, we confirmed eGFP expression by ELISA and *C9orf72* reduction by qPCR in brain and spinal cord of 3-month-old *C9orf72* eGFP knock-in mice (Extended Data Fig. 2c,d). These results confirm that our DPR knock-in mouse lines selectively express their specific DPR, in combination with *C9orf72* reduction, and that polyGR is expressed in the physiological range.

### PolyGR is predominantly expressed in neurons

**Fig. 2:**
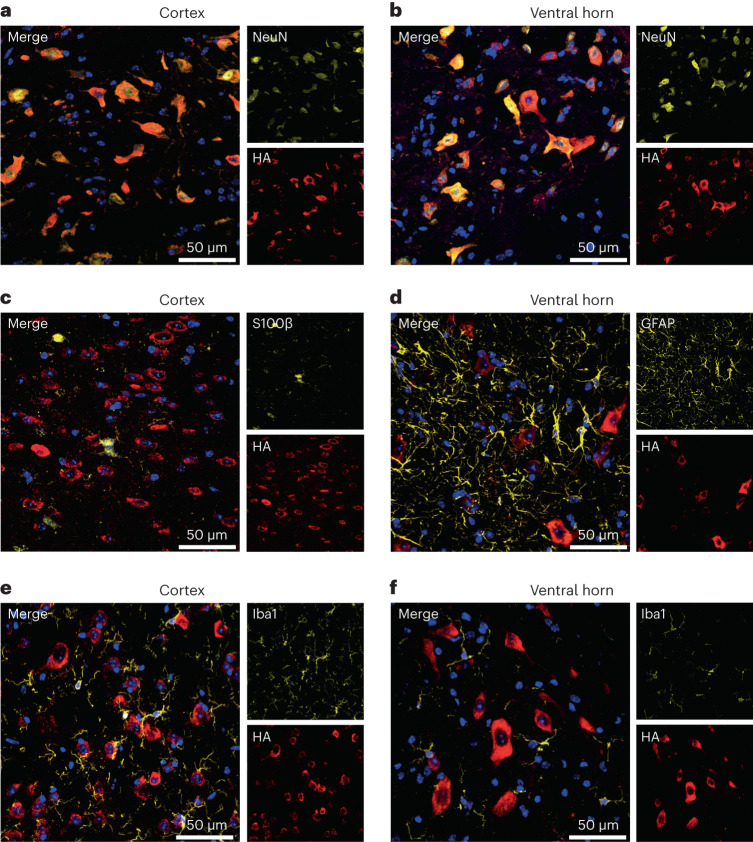
PolyGR is predominantly expressed in neurons. **a**,**b**, Representative confocal images of immunofluorescence staining showing colocalization between neuronal marker NeuN (yellow) and HA-tag (red) in (GR)400 mouse brain cortex (**a**) and lumbar spinal cord ventral horn (**b**) at 6 months of age, *n* = 5 mice per genotype. **c**,**d**, Representative confocal images of immunofluorescence staining showing absence of colocalization between the astrocytic markers S100β (**c**) or GFAP (**d**) (yellow) and HA-tag (red) in (GR)400 mouse brain cortex (**c**) and lumbar spinal cord ventral horn (**d**) at 6 months of age, *n* = 5 mice per genotype. **e**,**f**, Representative confocal images of immunofluorescence staining showing absence of colocalization between the microglial marker Iba1 (yellow) and HA-tag (red) in (GR)400 mouse brain cortex (**e**) and lumbar spinal cord ventral horn (**f**) at 6 months of age. DAPI (blue) stains nuclei, *n* = 5 mice per genotype.

Having established DPRs are translated in brain and spinal cord, we used the HA-tag to visualize them by immunostaining. PolyGR showed a clear cytoplasmic localization in brain and spinal cord at 6 months of age (Extended Data Fig. 2e). However, we did not observe polyPR staining with the same conditions using antibodies against the HA-tag or polyPR (Extended Data Fig. 2e,f), perhaps due to inability to detect the native conformation of polyPR in vivo. Therefore, we focused on characterizing polyGR in more detail: polyGR shows widespread neuronal expression, colocalizing with neuronal marker NeuN in cortex and in the ventral horn of lumbar spinal cord (Fig. 2a,b). At the same timepoint, we did not observe colocalization of polyGR with astrocytic S100β, GFAP, or microglial marker Iba1 (Fig. 2c–f). These results show that in our knock-in mice, polyGR is neuronally expressed in ALS/FTD relevant regions.

### (GR)400 knock-in mice exhibit cortical hyperexcitability

**Fig. 3:**
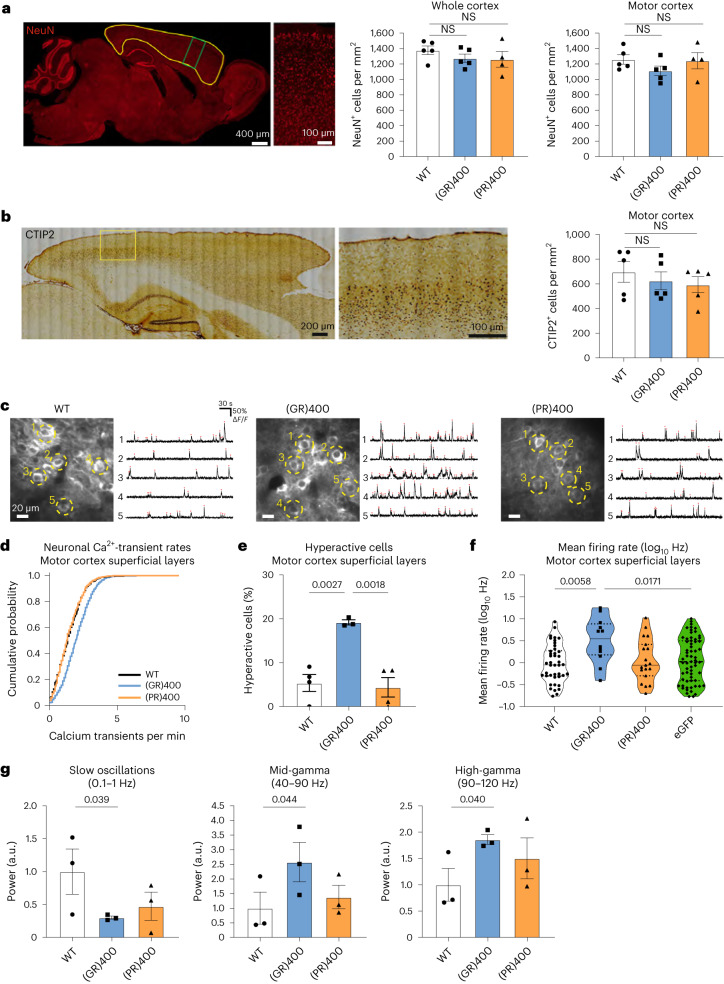
(GR)400 knock-in mice exhibit cortical hyperexcitability without neuronal loss. **a**, Representative image of the cortex (area delineated in yellow), enhanced magnification of the motor cortex (area delineated in green), and quantification of NeuN-positive (red) cell density in the whole cortex and in motor cortex in WT, (GR)400 and (PR)400 mice at 12 months of age. Graph, mean ± s.e.m., *n* = 4–5 mice per genotype, one-way ANOVA, Bonferroni’s multiple comparison. NS denotes *P* > 0.05. **b**, Representative image of the cortex, enhanced magnification of the motor cortex (area delineated in yellow) and quantification of CTIP2-positive upper motor neuron density in layer V of the motor cortex in WT, (GR)400 and (PR)400 mice at 12 months of age. Graph, mean ± s.e.m., *n* = 5 mice per genotype, one-way ANOVA, Bonferroni’s multiple comparison. NS denotes *P* > 0.05. **c**, Example of in vivo two-photon fluorescence images of jRCamP1b-expressing superficial layer neurons in motor cortex and spontaneous Ca^2+^ activity from five example neurons in WT (left panel), GR(400) (center panel) and PR(400) (right panel) mice at 15–19 months of age. **d**, Cumulative distribution plot displaying neuronal Ca^2+^-transient rates across animals in motor cortex superficial layers of WT (732 cells, 4 mice), (GR)400 (1,405 cells, 3 mice) and (PR)400 (946 cells, 4 mice) mice at 15–19 months of age. **e**, Percentage of hyperactive (>3 Ca^2+^-transients per minute) neurons in motor cortex superficial layers of WT (*n* = 4 mice), (GR)400 (*n* = 3 mice) and (PR)400 (*n* = 4 mice) mice at 15–19 months of age. Graph, mean ± s.e.m., one-way ANOVA, Tukey’s multiple comparison. **f**, Mean firing rate (log_10_Hz) of neurons in motor cortex superficial layers of WT, (GR)400, (PR)400 and eGFP mice at 15–19 months of age; black lines indicate medians and dashed lines indicate quartiles. Graph, *n* = 3 mice per genotype, one-way ANOVA, Tukey’s multiple comparison. **g**, LFP power in the slow-wave frequency band (left panel), mid gamma-frequency band (center panel) and high gamma-frequency band (right panel) in WT, (GR)400 and (PR)400 mice at 15–19 months of age. Graph, mean ± s.e.m., *n* = 3 mice per genotype, one-tailed one-way ANOVA, Fisher’s least significant difference procedure following Levene test for equal variances and test for normality. Source data

**Extended Data Fig. 3:**
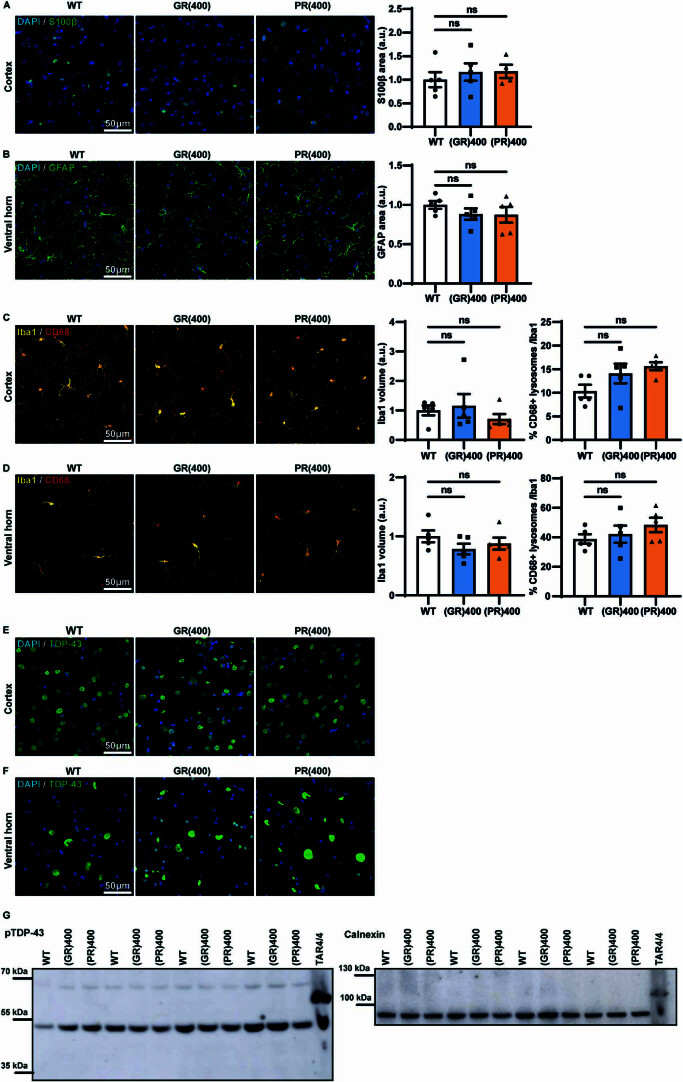
(GR)400 and (PR)400 knock-in mice do not exhibit gliosis in brain and spinal cord up to 12 months of age. (**a**) Representative confocal images and quantification of immunofluorescence staining of astrocytic marker S100β (green) and DAPI (blue) in brain cortex in WT, (GR)400, and (PR)400 mice at 12 months of age. Graph, mean ± SEM, n = 4-5 mice per genotype, one-way ANOVA, Bonferroni’s multiple comparison, ns for p > 0.05. (**b**) Representative confocal images and quantification of immunofluorescence staining of astrocytic marker GFAP (green) in lumbar spinal cord ventral horn in WT, (GR)400, and (PR)400 mice at 12 months of age. DAPI (blue) stains nuclei. Graph, mean ± SEM, n = 5 mice per genotype, one-way ANOVA, Bonferroni’s multiple comparison, ns denotes p > 0.05. (**c**) Representative confocal images and quantification of immunofluorescence staining showing microglial density and colocalization between microglial markers Iba1 (yellow) and microglial lysosomal marker CD68 (red) in brain cortex in WT, (GR)400, and (PR)400 mice at 12 months of age. Graph, mean ± SEM, n = 5 mice per genotype, one-way ANOVA, Bonferroni’s multiple comparison, ns denotes p > 0.05. (**d**) Representative confocal images and quantification of immunofluorescence staining showing microglial density and colocalization between microglial markers Iba1 (yellow) and microglial lysosomal marker CD68 (red) in lumbar spinal cord ventral horn in WT, (GR)400, and (PR)400 mice at 12 months of age. Graph, mean ± SEM, n = 5 mice per genotype, one-way ANOVA, Bonferroni’s multiple comparison, ns denotes p > 0.05. (**e**) Representative confocal images of immunofluorescence staining showing TDP-43 (green) cellular localisation in brain cortex in WT, (GR)400, and (PR)400 mice at 12 months of age. DAPI (blue) stains nuclei. n = 5 mice per genotype. (**f**) Representative confocal images of immunofluorescence staining showing TDP-43 (green) cellular localisation in lumbar spinal cord ventral horn in WT, (GR)400, and (PR)400 mice at 12 months of age. DAPI (blue) stains nuclei. n = 5 mice per genotype. (**g**) Western blotting analysis of phosphorylated TDP-43 protein levels in spinal cord of WT, (GR)400, and (PR)400 mice at 12 months of age shows no abnormal p-TDP-43 bands in the knock-in mice. Cortex of P24 homozygous TAR4/4 mice, which develop disease-associated pTDP-43 is used as positive control. Calnexin is shown as loading control. n = 4 mice per genotype. Source data

Next, we assessed whether polyGR or polyPR expression in the brain is associated with pathological features of ALS and FTD. We did not observe astrogliosis or microgliosis in the motor cortex of (GR)400 and (PR)400 mice up to 12 months of age (Extended Data Fig. 3a,c). TDP-43 localization was also not altered up to 12 months of age (Extended Data Fig. 3e). The density of NeuN-positive neurons in the whole cortex and specifically in the motor cortex was not altered in (GR)400 and (PR)400 mice at 12 months of age (Fig. 3a). CTIP2-positive upper motor neurons in layer V of the motor cortex, which are particularly vulnerable to cell death in ALS, were also not reduced in 12-month-old (GR)400 and (PR)400 mice (Fig. 3b). These results suggest that, up to 12 months of age, polyGR and polyPR expression in the brain is not associated with pathological features found in ALS and FTD, including astrogliosis, microgliosis, TDP-43 pathology and neuronal loss.

**Extended Data Fig. 4:**
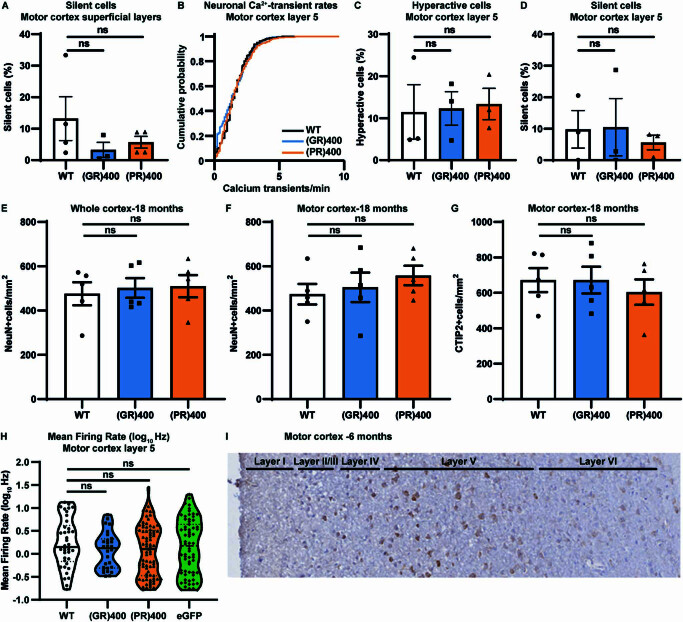
(GR)400 and (PR)400 knock-in mice do not exhibit cortical hyperexcitability in motor cortex layer 5. (**a**) Percentage of silent (0 Ca^2+^-transients per min) neurons in motor cortex superficial layers of WT (n = 4 mice), (GR)400 (n = 3 mice), and (PR)400 (n = 4 mice) mice at 15-19 months of age. Graph, mean ± SEM, one-way ANOVA, Tukey’s multiple comparison, ns denotes p > 0.05. (**b**) Cumulative distribution plot displaying neuronal Ca^2+^-transient rates across animals in layer 5 of WT (187 cells, 4 mice), (GR)400 (616 cells, 3 mice) and (PR)400 (305 cells, 4 mice) mice at 15-19 months of age. (**c**) Percentage of hyperactive (>3 Ca^2+^-transients per minute) neurons in motor cortex layer 5 of WT, (GR)400, and (PR)400 mice at 15-19 months of age. Graph, mean ± SEM, n = 3 mice per genotype, one-way ANOVA, Tukey’s multiple comparisons, ns denotes p > 0.05. (**d**) Percentage of silent (0 Ca^2+^-transients per min) neurons in motor cortex layer 5 of WT, (GR)400, and (PR)400 mice at 15-19 months of age. Graph, mean ± SEM, n = 3 mice per genotype, one-way ANOVA, Tukey’s multiple comparisons, ns denotes p > 0.05. (**e**) Quantification of NeuN-positive cell density in the whole cortex in WT, (GR)400, and (PR)400 mice at 18 months of age. Graph, mean ± SEM, n = 5 mice per genotype, one-way ANOVA, Bonferroni’s multiple comparison, ns denotes p > 0.05. (**f**) Quantification of NeuN-positive cell density in motor cortex in WT, (GR)400, and (PR)400 mice at 18 months of age. Graph, mean ± SEM, n = 5 mice per genotype, one-way ANOVA, Bonferroni’s multiple comparison, ns denotes p > 0.05. (**g**) Quantification of CTIP2-positive upper motor neuron density in layer V of the motor cortex in WT, (GR)400, and (PR)400 mice at 18 months of age. Graph, mean ± SEM, n = 5 mice per genotype, one-way ANOVA, Bonferroni’s multiple comparison, ns denotes p > 0.05. (**h**) Mean firing rate (log_10_Hz) of neurons in motor cortex layer 5 in WT, (GR)400, (PR)400, and eGFP mice at 15-19 months of age; black lines indicate medians, dashed lines indicate quartiles. Graph, n = 3 mice per genotype, one-way ANOVA, Tukey’s multiple comparison, ns denotes p > 0.05. (**i**) Representative image of the motor cortex showing HA-tag localisation form upper to lower cortical layers in (GR)400 mice at 6 months of age. n = 3 mice per genotype. Source data

We therefore investigated whether functional deficits were present, which would provide insight into the early brain changes in FTD and ALS. We conducted in vivo two-photon calcium imaging of neurons in superficial and deep layers of the motor cortex of (GR)400 and (PR)400 mice as well as wild-type (WT) controls using the red-shifted genetically encoded calcium indicator jRCaMP1b at 18 months of age (Fig. 3c). In (GR)400 mice we found an increase in the fraction of abnormally hyperactive neurons in superficial layers, but not in layer 5 where spontaneous neuronal activity was comparable to (PR)400 and WT mice (Fig. 3d,e and Extended Data Fig. 4a–d). We confirmed that there is no cortical neuronal loss at this timepoint, indicating a functional defect (Extended Data Fig. 4e–g). To further validate these layer-specific neuronal impairments, we performed high-density in vivo Neuropixels recordings in a subset of the same mice and in an additional cohort, including *C9orf72* eGFP knock-in mice, to assess single-unit and population neuronal activity. These experiments confirmed that motor cortex neurons in superficial layers in (GR)400 mice were hyperexcitable relative to those in (PR)400, WT and eGFP knock-in mice (Fig. 3f and Extended Data Fig. 4h). Furthermore, evaluation of population local field potential (LFP) across all motor cortex laminae suggested a concurrent reduction in slow-wave activity power, and an increase in gamma-frequency band power, in (GR)400 mice compared with (PR)400 and WT mice (Fig. 3g). These layer-specific changes were not due to layer-specific expression of polyGR as immunohistochemistry revealed polyGR expression across all cortical layers (Extended Data Fig. 4i). These findings provide evidence for augmented neuronal and network excitability in motor cortex of (GR)400 mice.

### (GR)400 and (PR)400 mice develop lower motor neuron loss

**Fig. 4:**
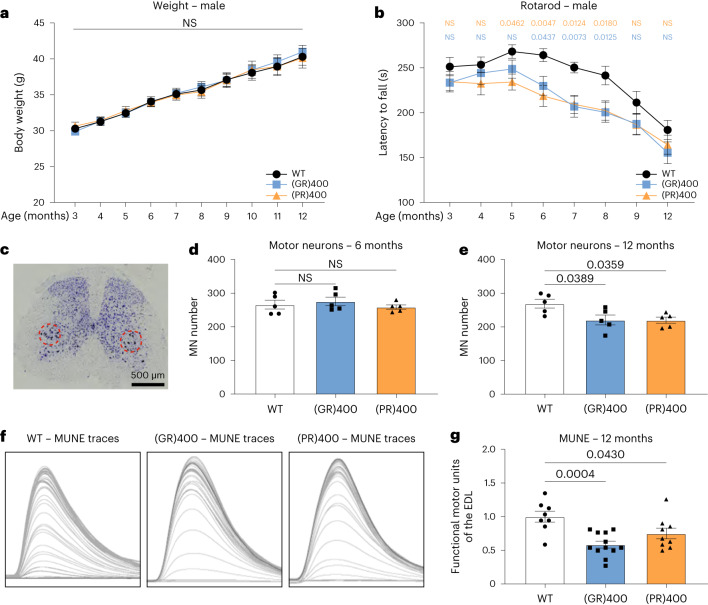
(GR)400 and (PR)400 knock-in mice develop age-dependent lower motor neuron loss and progressive rotarod impairment. **a**, Body weights of WT, (GR)400 and (PR)400 male mice up to 12 months of age. Graph, mean ± s.e.m., *n* = 14 mice per genotype, two-way ANOVA, Bonferroni’s multiple comparison. NS denotes *P* > 0.05. **b**, Accelerated rotarod analysis of motor coordination in WT, (GR)400 and (PR)400 male mice up to 12 months of age. Graph, mean ± s.e.m., *n* = 14 mice per genotype, two-way ANOVA, Bonferroni’s multiple comparison. NS denotes *P* > 0.05. **c**, Panel shows representative image of Nissl staining of lumbar spinal cord in WT mice; the dashed red line delineates the sciatic motor pool in which motor neurons were counted. *n* = 5 mice per genotype. **d**, Quantification of Nissl-stained motor neurons in lumbar spinal cord region L3–L5 in WT, (GR)400 and (PR)400 mice at 6 months of age. Graph, mean ± s.e.m., *n* = 5 mice per genotype, one-way ANOVA, Bonferroni’s multiple comparison. NS denotes *P* > 0.05. **e**, Quantification of Nissl-stained motor neurons in lumbar spinal cord region L3–L5 in WT, (GR)400 and (PR)400 mice at 12 months of age. Graph, mean ± s.e.m., *n* = 5 mice per genotype, one-way ANOVA, Bonferroni’s multiple comparison. **f**, Representative EDL MUNE traces from 12-month-old WT (left panel), (GR)400 (center panel) and (PR)400 (right panel) mice. Peaks correspond to physiological recruitment of motor units following electrical stimulation; *n* mice = 5 WT, 6 (GR)400 and 6 (PR)400; *n* muscles = 8 WT, 12 (GR)400 and 9 (PR)400. **g**, Quantification of MUNE determined in EDL muscle in WT, (GR)400 and (PR)400 mice at 12 months of age. Graph, mean ± s.e.m.; *n* mice = 5 WT, 6 (GR)400 and 6 (PR)400; *n* muscles = 8 WT, 12 (GR)400 and 9 (PR)400; one-way ANOVA, Bonferroni’s multiple comparison. MN, motor neuron. Source data

**Extended Data Fig. 5:**
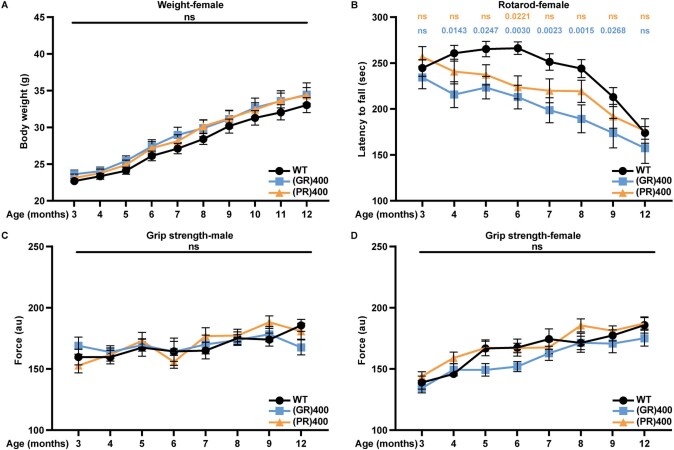
Female poly(GR) and poly(PR) knock-in mice develop rotarod impairment. (**a**) Body weights of WT, (GR)400, and (PR)400 female mice up to 12 months of age. Graph, mean ± SEM, n = 14 mice per genotype, two-way ANOVA, Bonferroni’s multiple comparison, ns denotes p > 0.05. (**b**) Accelerated rotarod analysis of motor coordination in WT, (GR)400, and (PR)400 female mice up to 12 months of age. Graph, mean ± SEM, n = 14 mice per genotype, two-way ANOVA, Bonferroni’s multiple comparison, ns denotes p > 0.05. (**c**) Grip strength analysis of muscle force in WT, (GR)400, and (PR)400 male mice over lifespan. Graph, mean ± SEM, n = 14 mice per genotype, two-way ANOVA, Bonferroni’s multiple comparison, ns denotes p > 0.05. (**d**) Grip strength analysis of muscle force in WT, (GR)400, and (PR)400 female mice over lifespan. Graph, mean ± SEM, n = 14 mice per genotype, two-way ANOVA, Bonferroni’s multiple comparison, ns denotes p > 0.05. Source data

**Extended Data Fig. 6:**
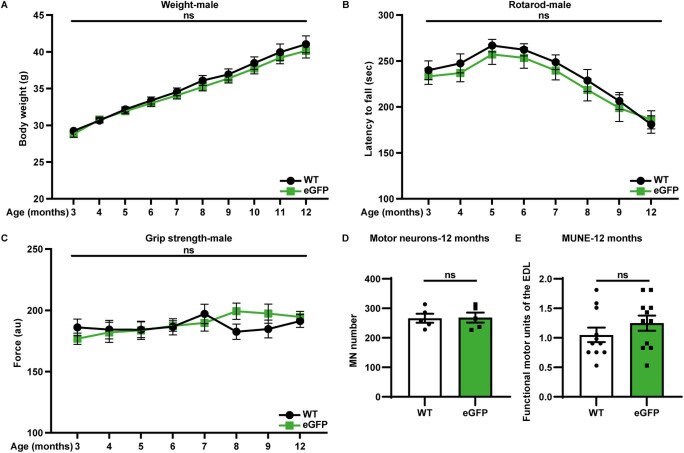
eGFP knock-in mice do not develop motor unit reduction or rotarod impairment. (**a**) Body weight analysis of WT and eGFP male mice over lifespan. Graph, mean ± SEM, n = 14 mice per genotype, two-way ANOVA, Bonferroni’s multiple comparison, ns denotes p > 0.05. (**b**) Accelerated rotarod analysis of motor coordination in WT and eGFP male mice from 3 to 12 months of age. Graph, mean ± SEM, n = 14 mice per genotype, two-way ANOVA, Bonferroni’s multiple comparison, ns denotes p > 0.05. (**c**) Grip strength analysis of muscle force in WT and eGFP male mice over lifespan. Graph, mean ± SEM, n = 14 mice per genotype, two-way ANOVA, Bonferroni’s multiple comparison, ns denotes p > 0.05. (**d**) Quantification of Nissl-stained motor neurons in lumbar spinal cord region L3-L5 in WT and eGFP mice at 12 months of age. Graph, mean ± SEM, n = 5 mice per genotype, two-sided unpaired two-sample Student’s t-test, ns denotes p > 0.05. (**e**) Quantification of MUNE determined in EDL muscle in WT and eGFP mice at 12 months of age. Graph, mean ± SEM, n mice = 6 WT and 6 eGFP, n muscles = 11 WT and 11 eGFP, two-sided unpaired two-sample Student’s t-test, ns denotes p > 0.05. Source data

Having identified functional deficits in the brain, we next assessed motor function in our DPR knock-in mice. Monthly body weight measurements showed no difference between (GR)400, (PR)400, eGFP and WT mice over the course of the first year of life (Fig. 4a and Extended Data Figs. 5a and 6a). Next, we evaluated motor coordination using the rotarod. Notably, we found a progressive decrease in accelerated rotarod performance in (GR)400 and (PR)400 mice (Fig. 4b and Extended Data Fig. 5b). In particular, (GR)400 mice showed significant rotarod impairment from 5 to 8 months of age, while in (PR)400 mice we found an impairment from 6 to 8 months of age, when compared with WT littermates, which showed an age-related decline from 8 months of age onwards, as expected. We did not observe rotarod deficits in eGFP knock-in mice over the first year of life when compared with WT littermates (Extended Data Fig. 6b), indicating the rotarod defect is a specific effect of DPR expression. We conducted grip strength analysis and neither (GR)400, nor (PR)400, nor eGFP knock-in mice showed strength deficits up to 12 months of age (Extended Data Figs. 5c,d and 6c). These results show that polyGR and polyPR cause progressive, but subtle, motor dysfunction, consistent with DPR expression levels within the physiological range.

We next turned our attention to neuropathological analysis of the ventral horn of the lumbar spinal cord as this is where the large alpha motor neurons reside that undergo degeneration in ALS. Similarly to the brain, we did not observe astrogliosis or microgliosis in the ventral horn of lumbar spinal cord from 12-month-old (GR)400 and (PR)400 mice (Extended Data Fig. 3b,d). Moreover, no signs of TDP-43 mis-localization, aggregation or altered phosphorylation were identified (Extended Data Fig. 3f,g).

**Extended Data Fig. 7:**
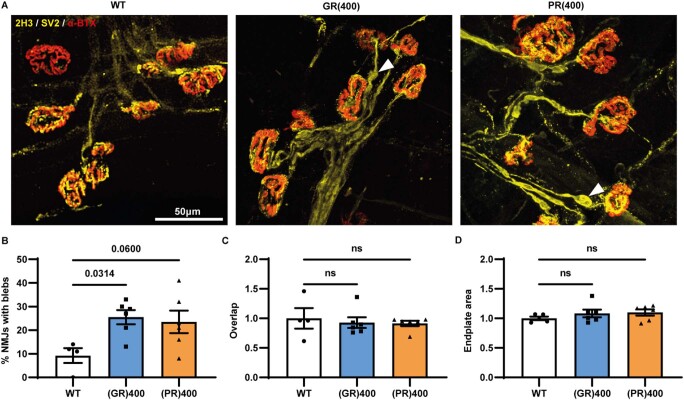
Neuromuscular junction analysis in hindlimb lumbrical muscles of WT, (GR)400, and (PR)400 mice. (**a**) Representative confocal images of hindlimb lumbrical muscles stained to visualize pre- (SV2A/2H3, yellow) and post-synaptic (α-Bungarotoxin, red) NMJ morphology in WT, (GR)400, and (PR)400 mice at 12 months of age. n = 4-6 mice per genotype. Arrowheads indicate blebbing events. (**b–d**) Quantification of blebbing events (**b**), pre- and post-synaptic overlap (**c**) and endplate area (**d**) in WT, (GR)400, and (PR)400 mice at 12 months of age. Graph, mean ± SEM, n = 4-6 mice per genotype, one-way ANOVA, Bonferroni’s multiple comparison, ns denotes p > 0.05. Source data

We assessed neurodegeneration by counting motor neurons in the lumbar spinal cord (Fig. 4c). We did not observe any difference in motor neuron numbers in 6-month-old (GR)400 and (PR)400 mice compared with WT mice (Fig. 4d). However, at 12 months of age, polyGR and polyPR mice showed a nearly 20% reduction in the number of motor neurons in the lumbar spinal cord (Fig. 4e), while eGFP knock-in mice showed no reduction (Extended Data Fig. 6d). This is an important result as it shows that our knock-in mice replicate a cardinal feature of ALS: age-dependent spinal cord motor neuron loss. Given the importance of these findings, we next used in vivo electrophysiological recordings to validate loss of motor neurons. We performed motor unit number estimation (MUNE) analysis in the hindlimbs of 12-month-old (GR)400 and (PR)400 mice. We detected a significant reduction, by 41% in (GR)400 and 25% in (PR)400, in functional motor unit number in the extensor digitorum longus (EDL) muscles when compared with WT littermates (Fig. 4f,g). Furthermore, we performed the same recordings in the hindlimbs of 12-month-old *C9orf72* eGFP knock-in mice without finding alterations in functional motor unit number (Extended Data Fig. 6e). Analysis of neuromuscular junctions (NMJs) in (GR)400 and (PR)400 mice at 12 months of age revealed an increase in axonal blebbing without gross denervation (Extended Data Fig. 7a–d). Overall, these results reveal an age-dependent neurodegeneration in the spinal cord of our polyGR and polyPR knock-in mouse models.

### Increased ECM proteins in (GR)400 and (PR)400 spinal cord

**Fig. 5:**
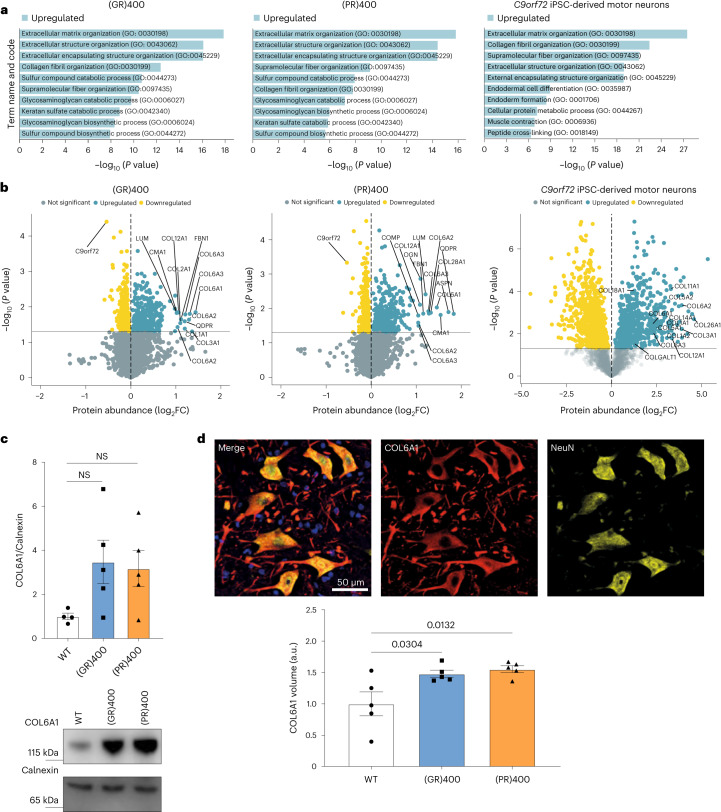
(GR)400 and (PR)400 knock-in mouse spinal cord has increased ECM protein levels. **a**, GO term enrichment analysis from significantly upregulated (blue) proteins in the lumbar spinal cord of 12-month-old (GR)400 (left panel) or (PR)400 (center panel) mice and *C9orf72* patient iPS cell-derived motor neurons (right panel). Proteomics performed on WT (*n* = 4 mice), (GR)400 (*n* = 5 mice), (PR)400 (*n* = 6 mice); two-sided Welch’s *t*-test with 5% FDR multiple-correction. **b**, Protein expression volcano plots from the lumbar spinal cord of 12-month-old (GR)400 (left panel) or (PR)400 (center panel) mice, and *C9orf72* patient iPS cell-derived motor neurons (right panel). WT (*n* = 4 mice), (GR)400 (*n* = 5 mice), (PR)400 (*n* = 6 mice); two-sided Welch’s *t*-test with 5% FDR multiple-correction. **c**, Western blot of COL6A1 in lumbar spinal cord of WT, (GR)400 and (PR)400 mice at 12 months of age. Calnexin is shown as loading control. Graph, mean ± s.e.m.; WT (*n* = 4 mice), (GR)400 (*n* = 5 mice), (PR)400 (*n* = 5 mice); one-way ANOVA, Bonferroni’s multiple comparison. NS denotes *P* > 0.05. **d**, Representative confocal images and quantification of immunofluorescence staining of COL6A1 (red) and NeuN (yellow) in lumbar spinal cord in WT mice at 12 months of age. DAPI (blue) stains nuclei. Graph, mean ± s.e.m., *n* = 5 mice per genotype, one-way ANOVA, Bonferroni’s multiple comparison. FC, fold change. Source data

**Extended Data Fig. 8:**
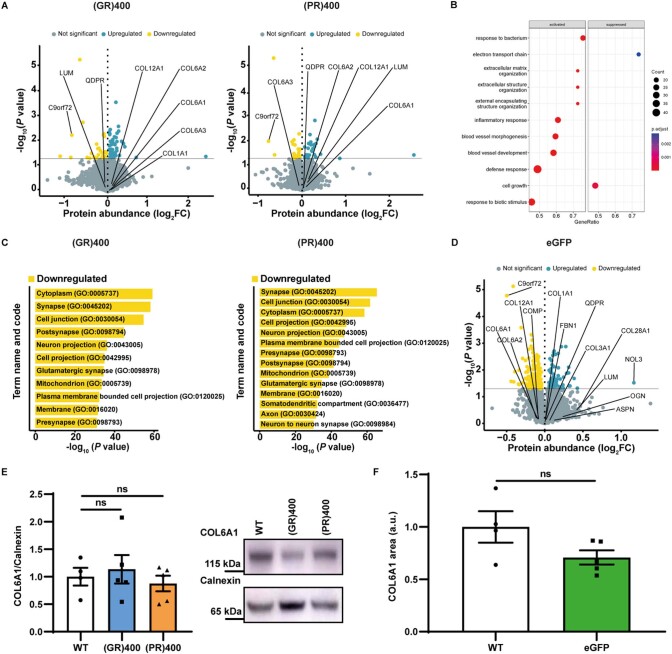
(GR)400 and (PR)400 knock-in mouse spinal cord extracellular matrix signature is conserved in *C9orf72* patient motor neurons and (GR)400 and (PR)400 knock-in mouse spinal cord have decreased synapse protein levels. (**a**) Protein expression volcano plots from the cortex of 12-month old (GR)400 (left panel) and (PR)400 (right panel) mice. WT (n = 4 mice), (GR)400 (n = 5 mice), (PR)400 (n = 5 mice), two-sided Welch’s t-Test with 5% FDR multiple-correction. (**b**) Significantly enriched activated (left) and suppressed (right) Gene Ontology (GO) pathways in *C9orf72* patient laser capture microdissected spinal cord motor neuron microarray data. The names of GO terms are shown on the y axis, and the gene ratio is shown on the x axis. The depth of the colour illustrates the adjusted p-value, adjusted p values were calculated by Kolmogorov–Smirnov test with Benjamini-Hochberg correction. The size of the circle in the graph corresponds to the size of the gene set. (**c**) GO analysis displaying GO term name and code from significantly downregulated (yellow) proteins in the lumbar spinal cord of (GR)400 (left panel), or (PR)400 (right panel) mice at 12 months of age. Proteomics performed on WT (n = 4 mice), (GR)400 (n = 5 mice), (PR)400 (n = 6 mice), two-sided Welch’s t-Test with 5% FDR multiple-correction. (**d**) Protein expression volcano plot from the lumbar spinal cord of 12-month old eGFP mice. n = 5 mice per genotype, two-sided Welch’s t-Test with 5% FDR multiple-correction. (**e**) Western blot of COL6A1 in cortex of WT, (GR)400, and (PR)400 mice at 12 months of age. Calnexin is shown as loading control. Graph, mean ± SEM, n = 4-5 mice per genotype, one-way ANOVA, Bonferroni’s multiple comparison, ns denotes p > 0.05. (**f**) Quantification of immunofluorescence staining of COL6A1 in lumbar spinal cord in WT and eGFP mice at 12 months of age. Graph, mean ± SEM, n = 4 WT and 5 eGFP mice, two-sided unpaired two-sample Student’s t-test, ns denotes p > 0.05. Source data

We next investigated dysregulated pathways using quantitative proteomics. Analysis of lumbar spinal cord and cortex of 12-month-old (GR)400 and (PR)400 mice revealed a striking increase in ECM terms specifically in the spinal cord (Fig. 5a and Extended Data Fig. 8a), with no other clearly upregulated pathways in spinal cord or brain. We obtained proteomics data from NeuroLINCS, which recently developed an integrated multi-omic analysis of induced pluripotent stem (iPS) cell-derived motor neurons from patients with C9ALS (ref. ^30^). We analyzed their raw mass spectrometry data using our own analysis pipeline to ensure an appropriate comparison with our knock-in mouse data and observed a remarkable similarity between datasets, which showed a common upregulation of Gene Ontology (GO) terms associated with the ECM, consistent with the original NeuroLINCS findings^30,31^ (Fig. 5a). Multiple ECM-associated proteins, including collagens, were highlighted as the most significantly upregulated proteins in lumbar spinal cord of (GR)400 and (PR)400 mice, and iPS cell-derived motor neurons (Fig. 5b). We also analyzed a published dataset of laser-capture microdissected motor neurons from spinal cord of patients with C9ALS (ref. ^32^) and again ECM terms were among the most significantly upregulated GO terms (Extended Data Fig. 8b), consistent with a previous report^31^. Fewer proteins were downregulated and with lower fold-changes than upregulated proteins, but, interestingly, GO term enrichment analyses revealed synapse proteins were reduced in lumbar spinal cord of (GR)400 and (PR)400 mice (Extended Data Fig. 8c), consistent with the observed motor neuron loss.

**Extended Data Fig. 9:**
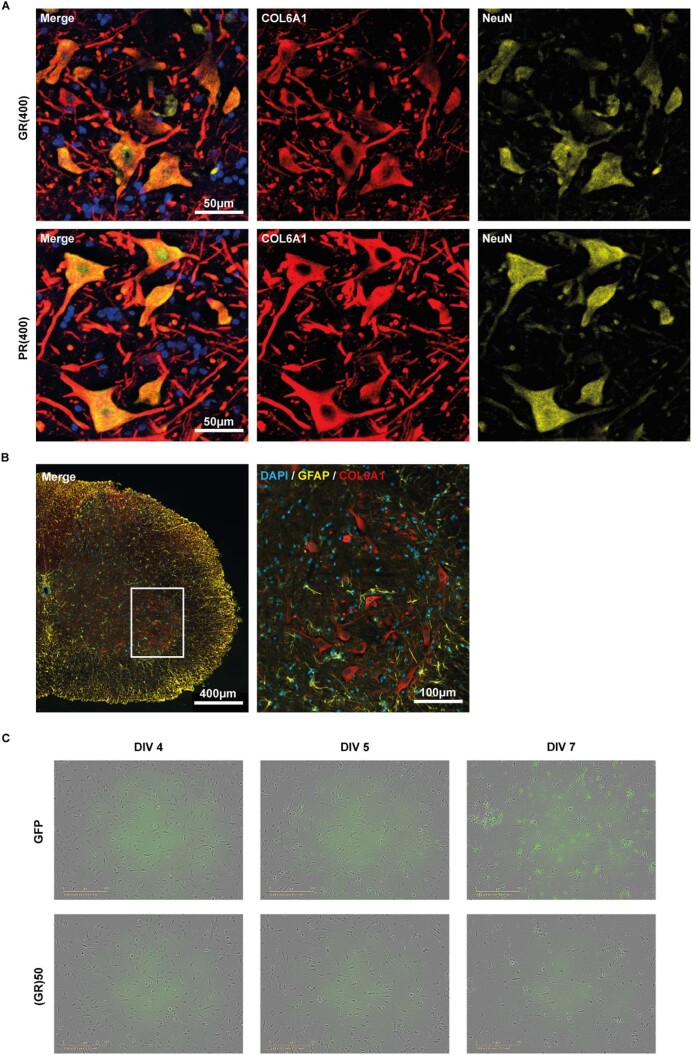
COL6A1 is predominantly expressed in neurons. (**a**) Representative confocal images of immunofluorescence staining of COL6A1 (red) and NeuN (yellow) in lumbar spinal cord in (GR)400, and (PR)400 mice at 12 months of age. DAPI (blue) stains nuclei. n = 5 mice per genotype. (**b**) Representative images of immunofluorescence staining of COL6A1 (red) and GFAP (yellow) in lumbar spinal cord in WT, (GR)400, and (PR)400 mice at 12 months of age. DAPI (blue) stains nuclei. n = 3 mice per genotype. (**c**) Representative Incucyte images of i^3^Neurons expressing (GR)_50_, or GFP at DIV 4, 5 and 7. n = 3 independent biological replicates.

To investigate whether ECM upregulation is associated with polyGR and polyPR expression or C9orf72 reduction, we performed quantitative proteomics on our eGFP knock-in mice. This analysis revealed that ECM-associated proteins were not altered in lumbar spinal cord of 12-month-old eGFP knock-in mice (Extended Data Fig. 8d). Importantly, a significant reduction in C9orf72 protein was observed in the knock-in mice, which is consistent with our qPCR and immunoblotting data (Fig. 5b and Extended Data Fig. 8d), confirming the quality of the dataset. To further verify our results, we evaluated one of the most altered ECM proteins in the proteomics analysis, COL6A1, in (GR)400 and (PR)400 mice. Immunoblot analysis showed COL6A1 was upregulated in lumbar spinal cord of 12-month-old (GR)400 and (PR)400 mice (Fig. 5c), but not in cortex (Extended Data Fig. 8e). Similarly, immunostaining followed by volumetric image analysis showed a ~50% increase of COL6A1 volume in the ventral horn of lumbar spinal cord from (GR)400 and (PR)400 mice at 12 months of age (Fig. 5d), but not eGFP knock-in mice (Extended Data Fig. 8f). Intriguingly, COL6A1 was localized to neurons rather than the extracellular space or other cell types (Extended Data Fig. 9a,b), indicating an increase of neuronal ECM protein expression. This is consistent with the increased ECM signature in patient iPS cell motor neurons and laser-capture microdissected motor neurons. Overall, these data show that an increase in ECM proteins, exemplified by COL6A1, is a conserved feature of C9ALS/FTD neurons.

### PolyGR induces TGF-β1 and its target gene *COL6A1* in i^3^Neurons

**Fig. 6:**
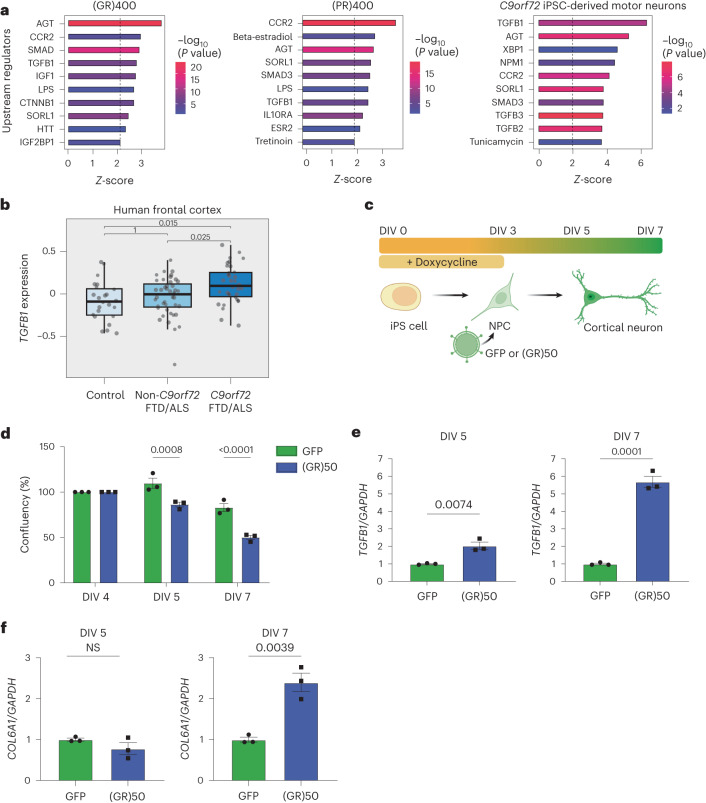
PolyGR induces *TGFB1* followed by its target gene *COL6A1* in i^3^Neurons. **a**, The top ten IPA-predicted upstream regulators in (GR)400 spinal cord (left panel), (PR)400 spinal cord (center panel) and *C9orf72* iPS cell-derived motor neurons (right panel). WT (*n* = 4 mice), (GR)400 (*n* = 5 mice), (PR)400 (*n* = 6 mice); two-sided Welch’s *t*-test with 5% FDR multiple-correction. **b**, Boxplot of residual gene expression for *TGFB1*. The boxplot midline represents the median, the box represents the interquartile range (25th and 75th percentiles) and the whiskers extend to the highest (upper whisker) or lowest (lower whisker) value, but no more than the interquartile range multiplied by 1.5. To determine differentially expressed genes, a linear regression model (two-sided) was used and *P* values were adjusted for multiple comparisons using the Benjamini–Hochberg FDR procedure. *C9orf72* FTLD/MND *n* = 34, non-*C9orf72* FTLD/MND *n* = 44, controls *n* = 24. **c**, Schematic workflow of i^3^Neuron doxycycline-induced differentiation at DIV 0 and transduction with lentiviruses expressing (GR)_50_, or GFP at DIV 3. **d**, Live-cell Incucyte confluency quantification in (GR)_50_, or GFP-treated i^3^Neurons. Graph, mean ± s.e.m., *n* = 3 independent biological replicates, two-way ANOVA, Bonferroni’s multiple comparison. **e**, qPCR analysis of *TGFB1* transcript levels normalized to *GAPDH* at DIV 5 (left panel) and DIV 7 (right panel) in (GR)_50_, or GFP-treated i^3^Neurons. Graph, mean ± s.e.m., *n* = 3 independent biological replicates, one-way ANOVA, Bonferroni’s multiple comparison. **f**, qPCR analysis of *COL6A1* transcript levels normalized to *GAPDH* at DIV 5 (left panel) and DIV 7 (right panel) in (GR)_50_, or GFP-treated i^3^Neurons. Graph, mean ± s.e.m., *n* = 3 independent biological replicates, one-way ANOVA, Bonferroni’s multiple comparison. NS denotes *P* > 0.05. FTLD, frontotemporal lobar degeneration. Source data

To identify potential upstream regulators controlling the differential expression of ECM-related proteins, we conducted Ingenuity Pathway Analysis (IPA) on the polyGR, polyPR, and patient iPS cell motor neuron datasets. IPA uncovered a number of predicted regulators. Several of the top predicted regulators were common between our DPR knock-in mice and human *C9orf72* iPS cell-derived motor neurons, including TGF-β1 and its intracellular mediator SMAD2/3, as well as AGT, CCR2 and SORL1 (Fig. 6a). Interestingly, among these regulators, we found that TGF-β1 signaling appeared significantly upregulated within the three proteomic datasets. To determine whether TGF-β1 is also altered in patient brain, we investigated a large frontal cortex RNA sequencing (RNA-seq) dataset comprising 34 C9ALS/FTD cases in which ECM dysregulation has also been reported^33^. We found that *TGFB1* was significantly increased, after genome-wide false discovery rate (FDR) correction, in C9ALS/FTD cases when compared with either non-C9ALS/FTD or neurologically normal controls (Fig. 6b), further confirming the relevance of this pathway in C9ALS/FTD patient tissue.

Based on these results, we hypothesized that activation of the TGF-β1 signaling pathway, which is known to be a master regulator of ECM genes^34^, may contribute to the ECM alterations in (GR)400 and (PR)400 mice. To test this hypothesis, we studied TGF-β1 signaling in vitro. We adopted a transcription factor-mediated differentiation protocol to differentiate human iPS cells into cortical neurons (i^3^Neurons)^35^. We transduced i^3^Neurons with 50 polyGR repeats ((GR)_50_)^36^, or with GFP as a negative control (Fig. 6c). (GR)_50_ caused a progressive increase in neuronal death, with a ~20% and ~40% reduction in confluency at 5 and 7 days in vitro (DIV), respectively, compared with GFP-treated cells (Fig. 6d and Extended Data Fig. 9c). qPCR analysis showed that *TGFB1* was significantly upregulated by polyGR at DIV 5, before its target gene *COL6A1*, which became significantly increased 2 days later (Fig. 6e,f). Thus, polyGR expression in neurons is sufficient to induce TGF-β1 expression, leading to increased expression of *COL6A1*, the most prominently increased ECM protein in polyGR knock-in mouse spinal cord. This suggests that increased TGF-β1 signaling contributes to the conserved ECM protein signature in C9ALS/FTD neurons.

### TGF-β1 and COL6A1 are neuroprotective in C9ALS/FTD models

**Fig. 7:**
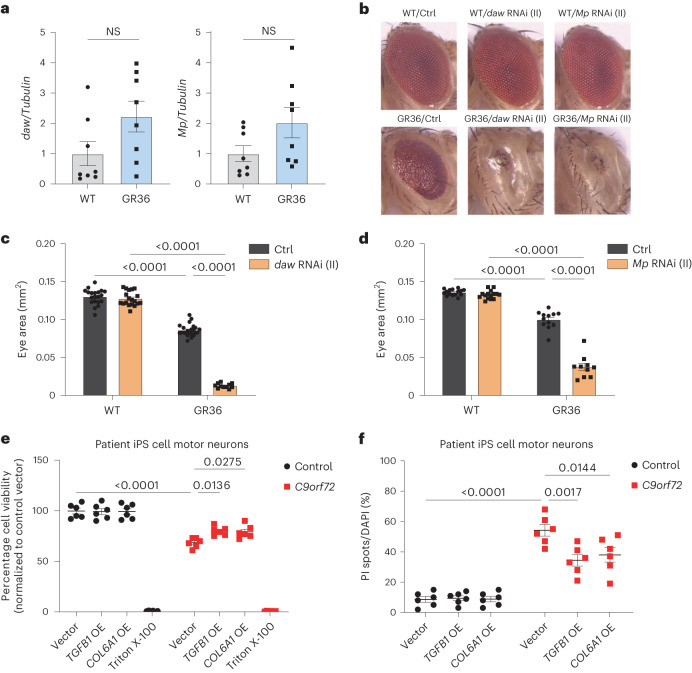
*TGFB1* and *COL6A1* are neuroprotective in C9ALS/FTD models. **a**, qPCR analysis of daw (left panel) and Mp (right panel) transcript levels normalized to Tubulin in WT and GR36 flies. Graph, mean ± s.e.m., *n* = 8 independent biological replicates, unpaired two-sample Student’s *t*-test. Genotypes: w; GMR-Gal4/+, w; GMR-Gal4, UAS-GR36/+. NS denotes *P* > 0.05. **b**, Stereomicroscopy images of representative 2-day-old adult WT (top panel) or GR36 (bottom panel) *Drosophila* eyes in absence of or co-expressing *daw* or *Mp* RNAi constructs inserted into the second chromosome (II). Genotypes: w; GMR-Gal4/+, w; GMR-Gal4/UAS-daw RNAi, w; GMR-Gal4/UAS-Mp RNAi, w; GMR-Gal4, UAS-GR36/+, w; GMR-Gal4, UAS-GR36/UAS-daw RNAi, w; GMR-Gal4, UAS-GR36/UAS-Mp RNAi. **c**, Eye size of flies normalized to the mean of the control eye size. Graph, mean ± s.e.m.; *n* (independent biological replicates, one eye counted per fly) = 20 WT/+, 19 WT/daw RNAi, 23 GR36/+, 11 GR36/daw RNAi; two-way ANOVA, Bonferroni’s multiple comparison. Genotypes: w; GMR-Gal4/+, w; GMR-Gal4/UAS-daw RNAi, w; GMR-Gal4, UAS-GR36/+, w; GMR-Gal4, UAS-GR36/UAS-daw RNAi. **d**, Eye size of flies normalized to the mean of the control eye size. Graph, mean ± s.e.m.; *n* (independent biological replicates, one eye counted per fly) = 15 WT/+, 15 WT/Mp RNAi, 12 GR36/+, 10 GR36/Mp RNAi; two-way ANOVA, Bonferroni’s multiple comparison. Genotypes: w; GMR-Gal4/+, w; GMR-Gal4/UAS-Mp RNAi, w; GMR-Gal4, UAS-GR36/+, w; GMR-Gal4, UAS-GR36/UAS-Mp RNAi. **e**, Percentage cell viability as measured by Alamar Blue following 4-h exposure to 10 µM glutamate. *n* = 6 control and 6 *C9orf72* iPS cell lines. Data points represent average percentage viability from three replicate wells for each condition. Two-way ANOVA with Tukey’s multiple comparison test was used to calculate statistical significance. **f**, Quantification of the ratio of PI-positive spots to DAPI-positive nuclei (quantification of cell death) following 4-h exposure to 10 µM glutamate. *n* = 6 control and 6 *C9orf72* iPS cell lines. Data points represent average percentage cell death across ten images per well. Two-way ANOVA with Tukey’s multiple comparison test was used to calculate statistical significance. Source data

**Extended Data Fig. 10:**
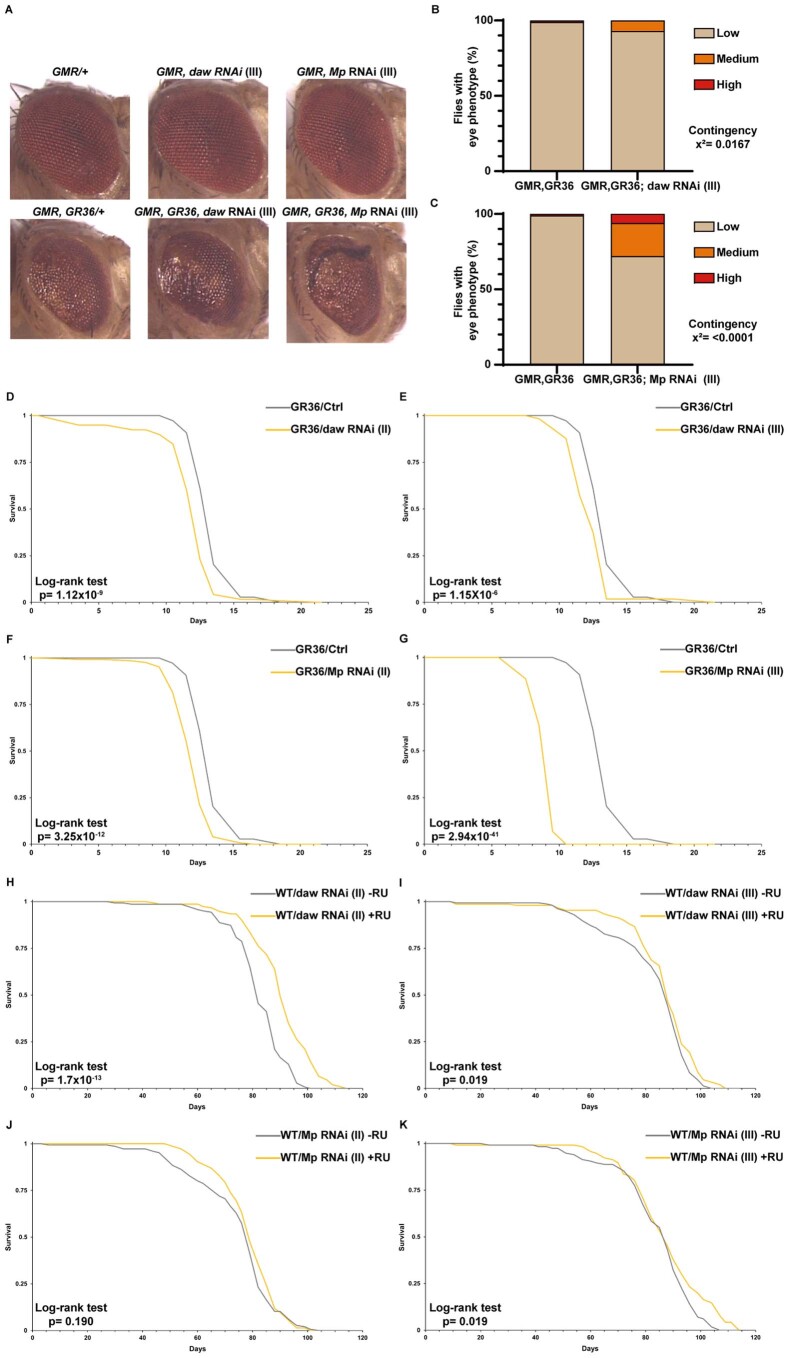
TGF-β1 and COL6A1 reduction specifically exacerbates polyGR toxicity *in vivo*. Two distinct RNAi lines per gene (*Mp* and *daw*) were tested, with the RNAi cassette inserted into either the attp40 or attp2 sites on the second (II) or third (III) chromosome respectively. (**a**) Stereomicroscopy images of representative 2-day old adult GMR (top panel) or GMR, GR36 (bottom panel) *Drosophila* eyes in absence or co-expressing daw or Mp RNAi constructs. Genotypes: GMR/+; GMR, *daw* RNAi (III); GMR, *Mp* RNAi (III); GMR, GR36/+; GMR, GR36, *daw* RNAi (III); GMR, GR36, *Mp* RNAi (III). (**b-c**) Quantification of the effects of *daw* and *Mp* RNAi on the rough eye phenotype in GR36 flies. n = 71 GMR,GR36, 45 GMR,GR36; *daw* RNAi (III), 68 GMR,GR36; *Mp* RNAi (III). The p value was determined by chi-squared test. (**d–g**) Lifespan of GR36/Ctrl (108), or GR36/*daw* RNAi (II) (118), or GR36/*daw* RNAi (III) (117), or GR36/*Mp* RNAi (II) (126), or GR36/*Mp* RNAi (III) (44). The p value was determined by log-rank test. Genotypes: w; UAS-(GR)36/+; ElavGS/+, w; UAS-(GR)36/UAS-*daw* RNAi; ElavGS/+, w; UAS-(GR)36/+; ElavGS/UAS-*daw* RNAi, w; UAS-(GR)36/UAS-*Mp* RNAi; ElavGS/+, w; UAS-(GR)36/+; ElavGS/UAS-*Mp* RNAi. (**h**) Lifespan of WT/*daw* RNAi (II) -RU (142) or WT/*daw* RNAi (II) + RU (153). The p value was determined by log-rank test vs. the vehicle-treated (-RU) group. Genotype: w; UAS-*daw* RNAi/+; ElavGS/+. (**i**) Lifespan of WT/*daw* RNAi (III) -RU (157) or WT/*daw* RNAi (III) + RU (153). The p value was determined by log-rank test vs. the vehicle-treated (-RU) group. Genotype: w; ElavGS/UAS-*daw* RNAi. (**j**) Lifespan of WT/*Mp* RNAi (II) -RU (146) or WT/*Mp* RNAi (II) + RU (146). The p value was determined by log-rank test vs. the vehicle-treated (-RU) group. Genotype: w; UAS-*Mp* RNAi/+; ElavGS/+. (**k**) Lifespan of WT/*Mp* RNAi (III) -RU (116) or WT/*Mp* RNAi (III) + RU (116). The p value was determined by log-rank test vs. the vehicle-treated (-RU) group. Genotype: w; ElavGS/UAS-*Mp* RNAi. Source data

The neuronal increase in ECM proteins could be protective, deleterious or neutral to disease progression. To investigate this further we focused on TGF-β1 and COL6A1. To determine whether TGF-β1 and COL6A1 have a role in polyGR-induced neurodegeneration in vivo, we utilized our *Drosophila* line expressing 36 polyGR repeats^7,37^, which causes a moderate eye degeneration. We investigated the *dawdle* (*daw*) and *Multiplexin* (*Mp*) genes, which are the closest fly orthologues of *TGFB1* and *COL6A1*, respectively. Both *daw* and *Mp* levels were increased in GR36 flies (Fig. 7a), consistent with both the knock-in mouse and patient iPS cell motor neuron data. We crossed GR36 flies with two different RNA interference (RNAi) lines targeting *daw* or *Mp*. Both *daw* and *Mp* reduction caused a worsening of eye degeneration in GR36 flies but had no effect in WT flies (Fig. 7b–d and Extended Data Fig. 10a–c). To assess their effect in adult neurons, we used the inducible neuronal elav-GS driver for expression of *daw* or *Mp* RNAi, combined with GR36, after eclosion, and monitored survival. Reduction of *daw* and *Mp* reduced survival of GR36 flies but not WT flies (Extended Data Fig. 10d–k). This shows that *daw* and *Mp* are able to specifically protect against polyGR-induced toxicity in vivo, indicating that increased neuronal collagen expression is neuroprotective in the context of polyGR insult. We next investigated whether TGFB1 or COL6A1 overexpression could be neuroprotective in iPS cell motor neurons. iPS cell motor neurons from patients with C9ALS/FTD have been shown to be more sensitive than control neurons to glutamate-induced cell death^28,38^. Using this paradigm, expression of TGFB1 or COL6A1 significantly reduced glutamate-induced excitotoxicity in iPS cell-derived motor neurons of patients with *C9orf72* repeat expansions (Fig. 7e,f), consistent with a neuroprotective function.

In summary, ECM proteins, exemplified by COL6A1, are specifically increased in the spinal cord of our new DPR knock-in mice, as well as in *Drosophila* and human iPS cell neurons expressing polyGR, patient iPS cell motor neurons and patient end-stage spinal motor neurons. The presence of this conserved signature in surviving neurons across different models and patient material, combined with our data showing a protective role in GR36 *Drosophila* and patient iPS cell-derived motor neurons, indicates a novel neuroprotective role of neuronally expressed ECM proteins.

## Discussion

We have generated DPR knock-in mice, using the endogenous mouse *C9orf72* promoter to drive expression of a single DPR, either (GR)400 or (PR)400. The insertion of the DPR sequence also removed one normal allele of *C9orf72*. Thereby we have recapitulated two features of C9ALS/FTD—the reduced level of C9orf72 and the presence of DPRs. The mice do not recapitulate the unconventional RAN translation mechanism of DPR generation, as they are driven by the mouse *C9orf72* ATG start codon. This was intentional, allowing us to study the effects of specific DPRs, and means that other mouse models, expressing the expanded G_4_C_2_ repeat, are needed to study mechanisms of RAN translation in vivo. We focused on polyGR and polyPR as they are consistently the most toxic DPRs in model systems^16^. This does not lessen the relevance of other DPRs, particularly polyGA, which is also toxic in different model systems, but likely through different mechanisms^3^.

We show that driving expression with the endogenous mouse promoter leads to relatively physiological expression levels of polyGR, as determined by comparison with human brain, allowing the examination of patient-relevant polyGR effects. It is important to note that differences between our knock-in mice and patient material make it hard to directly assess how comparable DPR expression levels are in the two systems. For instance, we analyzed bulk tissue, so the relative DPR expression level per neuron was not determined. The use of ATG-driven translation, rather than RAN translation, in our models would likely lead to higher translation efficiency and thus potentially higher DPR levels in our mice. The similarity in expression between our polyGR mice and human material may be due to comparing early/mid-stage tissue in the mice with end-stage human tissue, in which DPRs have had time to build up. Nonetheless, our data indicate that expression levels of polyGR are at least in the physiological range. PolyPR is harder to model physiologically as the promoter driving polyPR expression from the antisense strand is not known. We therefore used the endogenous mouse *C9orf72* sense strand promoter. This allows direct comparison with our polyGR mice but makes the polyPR model less physiological. As polyPR is less abundant than polyGR in human postmortem tissue, our polyPR mice, while still expressing at relatively low levels, are likely to express polyPR at higher levels than that observed in patients.

We also built DPR constructs in the patient range, comprising 400 uninterrupted polyGR or polyPR repeats, to replicate patient DPRs as closely as possible. Expression of patient-length repeats did not lead to overt gliosis or TDP-43 mis-localization over 12 months, nor did it lead to cortical neuronal loss. We observed a consistent deficit in rotarod performance in both polyPR and polyGR mice from 6 to 9 months of age, but this appeared transient as the WT mice started declining with age until they caught up and there was no longer a difference. We interpret this as polyGR and polyPR causing neuronal dysfunction early on but this being superseded by age-related decline in motor function. These results are consistent with the milder effects observed in knock-in mice, in which genes are expressed at more physiological levels, and provide reassurance that overexpression artefacts are unlikely^26^.

Intriguingly, using two complementary methods, namely two-photon calcium imaging and high-density Neuropixels recording, we observed hyperexcitability in polyGR mice in superficial cortical layers, which are affected in FTD, but not deeper layer 5 neurons which are vulnerable in ALS. Further research is needed to parse this difference and its relevance, but it is noteworthy that cortical hyperexcitability is well described in both *C9* and non-*C9* ALS/FTD patients^39^, indicating that new mechanistic insights into this early patient-relevant phenotype can be gained by future investigations in our polyGR mice. It is also interesting that only polyGR but not polyPR mice showed this phenotype. This points to a more pertinent effect of polyGR in the brain, consistent with several studies that show polyGR is the only DPR to correlate with clinical symptoms and neurodegeneration in patients with C9ALS/FTD (refs. ^40–42^). We cannot rule out that polyPR is expressed in different neuronal populations from polyGR as we have been unable to visualize polyPR using immunostaining techniques, although this is unlikely as polyPR is driven by the same promoter as polyGR so should be expressed in the same cells. Our inability to immunolocalize polyPR may be due to using antibodies raised against short peptides, which likely recognize short linear epitopes, rather than the native polyPR structure. The anti-polyPR antibody we used can recognize polyPR aggregates^43^, indicating that polyPR is not aggregated, but present in its native soluble form. Reassuringly, polyPR transcript levels are equivalent to polyGR and we detected denatured polyPR protein using Meso Scale Discovery (MSD) immunoassay in polyPR brain and spinal cord.

While we did not observe neuronal loss in the cortex, we did observe a significant, approximately 20% loss of spinal cord motor neurons in both polyGR and polyPR mice. We therefore focused our mechanistic analyses on the spinal cord. A striking finding was a remarkably conserved signature of increased ECM gene expression in C9ALS/FTD tissues. The increased ECM signature was the dominant change in quantitative proteomics analyses of both polyGR and polyPR knock-in spinal cord and it was remarkably similar to an independent quantitative proteomics dataset of *C9orf72* patient iPS cell motor neurons. This increase was also present in laser-capture microdissected *C9orf72* patient spinal cord motor neurons, human iPS cell neurons treated with polyGR and *Drosophila* overexpressing polyGR. This conservation across several models and human tissue indicates a genuine phenomenon linked to the presence of arginine-rich DPRs. Altered ECM gene expression has been noted in a range of ALS-related transcriptomic datasets both with and without *C9orf72* mutation, but there is no clear consensus on its role and importance in ALS pathophysiology. It is clear that one driver of increased ECM gene expression in those datasets is astrogliosis. A meta-analysis of human iPS cell astrocyte transcriptomic datasets from several genetic subtypes of ALS (*C9orf72*, *FUS*, *SOD1* and *VCP*) revealed a common increase in ECM gene expression that was shared with pro-inflammatory ‘A1’ astrocytes^44^. This is consistent with recently published bulk spinal cord RNA-seq data from a large series of 154 ALS cases (including 29 *C9orf72* cases^45^). Weighted gene co-expression network analysis of this dataset identified 23 co-expressed gene modules, including an astrocyte module with significantly increased ECM gene expression in ALS cases that was negatively correlated with age at onset and age at death^45^. This suggests that pro-inflammatory astrocytes are characterized by increased ECM gene expression and that this correlates with more severe disease. Therefore, ECM signals in bulk transcriptomic data could derive from both activated astrocytes and microglia, as well as neurons. Here we show a neuron-centric alteration in the absence of overt gliosis; however, these studies warrant future careful examination of the role of glia in ECM changes in C9ALS/FTD.

Our data now bring clarity to the complexities of ECM alterations in ALS/FTD by showing that there is a neuron-derived ECM signature that is neuroprotective. This provides a new paradigm for understanding ECM changes in which a potentially deleterious ECM increase in glia occurs alongside a protective ECM upregulation in neurons. These opposing effects may help explain why it has previously been hard to pinpoint the role and relevance of ECM alterations in ALS/FTD. The case for neuron-derived aberrant ECM expression is supported by its presence in neuronal datasets—patient iPS cell motor neurons and laser-capture microdissected motor neurons, as well as increased COL6A1 immunostaining in motor neurons in polyGR and polyPR knock-in mice. This ECM signature appears to be driven, at least in part, by TGF-β1. TGF-β1 is neuroprotective both in vitro and in vivo against a wide array of neuronal insults including excitotoxicity^46–48^, hypoxia^49^ and ischemia^50,51^. We now show TGF-β1 expression can lead to a neuroprotective increase in the neuronal expression of ECM proteins and specifically collagen VI.

Expression of polyGR in human iPS cell neurons was sufficient to induce *TGFB1* expression followed by *COL6A1*, and knockdown of the *COL6A1* or *TGFB1* homologs in flies expressing 36 GR repeats showed a specific and striking enhancement of neurodegeneration, pointing to a neuroprotective effect of collagen induction. In remarkable concordance with our data, it was previously shown that Aβ_42_ treatment of primary mouse neurons causes an increase in neuronal COL6A1 that is neuroprotective and mediated by TGF-β1 (ref. ^52^). Neuronal collagen VI expression is also increased upon ultraviolet-irradiation of primary neurons and protects against irradiation-induced apoptosis^53^. In combination, these results show that *COL6A1* is induced in neurons by neurodegenerative insults as a compensatory response, via TGF-β1. ECM proteins were not increased in the cortex of our knock-in mice, where we do not observe neuronal loss. This supports the concept of stress-induced TGF-β1 activation and protective ECM expression occurring downstream of an initial degenerative event. Collagen VI is composed of a 1:1:1 polymer of three distinct collagen VI chains encoded by *COL6A1*, *COL6A2* and *COL6A3*. This heterotrimer then tetramerizes before being secreted into the extracellular space where it classically forms beaded microfilaments^54^. However, in neuronal cultures collagen VI was identified in proximity to the neuronal plasma membrane, indicating that it may act as a neuroprotective autocrine signaling molecule when secreted by neurons rather than forming microfilaments^55^. Further investigations are now needed to identify the mechanism by which collagen VI provides neuroprotection.

It is clear that a range of different insults can induce TGF-β1/ECM changes. One such insult is polyGR/polyPR, but it is likely that other ALS-related insults can also lead to TGF-β1 and collagen increase. Indeed, dysregulated TGF-β1 signaling has been described in a *SOD1* mouse model of ALS^56–58^ and increased ECM and TGF-β1 signaling were identified in a transcriptomic dataset of sporadic ALS postmortem spinal motor neurons^31^. This indicates that different insults relevant to genetic or sporadic forms of ALS can cause a TGF-β1 response. In summary, these findings identify a neuroprotective neuronal ECM signature in C9ALS/FTD, exemplified by collagen VI, which may have broad relevance for ALS and other neurodegenerative diseases.

## Methods

### Assembly of targeting constructs

eGFP sequence and 100 codon-optimized polyGR or polyPR repeats were synthesized (Thermo Fisher Scientific). A pMC cloning vector was synthesized to contain 600 base pairs (bp) of the 5′ homology arm, a double HA-tag, a V5 epitope tag, an SV40 polyA tail and 250 bp of the 3′ homology arm. eGFP and 100 codon-optimized DPRs were cloned into this vector within the *BbsI* and *BsmBI* sites to generate pMC-eGFP, pMC-(GR)100 and pMC-(PR)100. Then, 400 codon-optimized DPRs were assembled with two consecutive rounds of recursive directional ligation taking advantage of the restriction enzymes *BbsI* and *BsmBI* to generate pMC-(GR)400 and pMC-(PR)400.

A selection cassette (FRT-PGK-gb2-neo-FRT, Gene Bridges) was inserted in the pMC-eGFP, pMC-(GR)400 and pMC-(PR)400 in the *NheI* site. A BAC subcloning kit (Red/ET recombination, Gene Bridges) was used to clone full-length homology arms (2.7 kilobases (kb) in 5′ and 3.2 kb in 3′) from the BAC clone (RP23-434N2), containing the C57BL/6J sequence of the mouse *C9orf72* gene, into the targeting vector pBlueScript II SK (+). Knock-in constructs were obtained by inserting sequences from pMC-eGFP, pMC-(GR)400 and pMC-(PR)400 into targeting vectors within the *BstXI* and *XcmI* sites.

### Animals

All procedures involving mice were conducted in accordance with the Animal (Scientific Procedures) Act 1986 and the Animal Research: Reporting of In Vivo Experiments guidelines and were performed at University College London (UCL) under an approved UK Home Office project license reviewed by the Institute of Prion Diseases Animal Welfare and Ethical Review Body. Mice were maintained in a 12-h light/dark cycle at a temperature of 20–24 °C and relative humidity of 45–55% with food and water supplied ad libitum. The knock-in mice generated are available from the European Mutant Mouse Archive, strain numbers (GR)400: EM:14658, (PR)400: EM:14659, eGFP: EM:15241.

To generate the DPR knock-in mouse strains, we performed CRISPR-assisted gene targeting in JM8F6 embryonic stem cells (C57BL/6N) using our targeting vector(s) and a CRISPR–Cas9 designed against the insertion site. The CRISPR construct (pX330-Puro-C9orf72) expressed Cas9 and a U6 promoter-driven single-guide RNA designed against the following sequence: AGTCGACATCCCTGCATCCC. This was generated by annealing two oligos (5′-CACCgAGTCGACATCCCTGCATCCC-3′; 5′-AAACGGGATGCAGGGATGTCGACTc-3′) and cloning this into the unique *BbsI* sites of pX330-U6-Chimeric_BB-CBh-hSpCas9 (Addgene no. 42230), modified by the addition of a PGK-Puro cassette. Then, 1 × 10^6^ embryonic stem cells were electroporated with 2.5 µg of the cloned pX330-Puro-C9orf72 plasmid and 2.5 µg of targeting vector using the Neon Transfection System (Thermo Fisher Scientific) (3 × 1,400 V, 10 ms) and plated on puromycin-resistant fibroblast feeder layers. After approximately 24 h, 600 ng ml^−1^ puromycin was applied for a further 48 h to allow transient selection. After a further 5 d in culture without selection, individual colonies were isolated, expanded and screened for the desired targeting at both the 5′ end (5′-TCGGGGATTATGCCTGCTGC’3′ and 5′-GCATCCCAGGTCTCACTGCA-3′) and the 3′ end (5′-TCGAAAGGCCCGGAGATGAGGAAG-3′ and 5′-GGGTTCAGACAGGTACAGCAT-3′). Embryonic stem cells from correctly targeted clones were injected into albino C57BL/6J blastocysts and the resulting chimeras were mated with albino C57BL/6J females. The presence of the targeted allele in the F1 generation was confirmed at the DNA level by the above PCR and Sanger sequencing. Germline-transmitting founders were obtained and backcrossed to WT C57BL/6J mice to maintain hemizygous lines.

Mouse genotype was determined by PCR for knock-in sequence with the following set of primers: forward 5′-TAAGCACAGCAGTCATTGGA-3′ and reverse 5′-AAGCGTAATCTGGAACATCG-3′. Repeat length was determined by PCR with the following set of primers: forward 5′-CCCATACGATGTTCCAGATTACGCTTACCC-3′ and reverse 5′-GCAATAAACAATTAGGTGCTATCCAGGCCCAG-3′.

Males were used for all experiments in the main text, except in vivo two-photon calcium imaging and Neuropixels recording where females were used. Phenotyping was also performed on female mice, with similar results to males, and these data are included in Extended data figures.

Homozygous TAR4/4 mice overexpressing WT human TARDBP (TDP-43)^59^ were used as a positive control for detecting phosphorylated TDP-43.

### Human tissues

Human *C9orf72* ALS/FTD samples for polyGR MSD immunoassay were described previously^42^ and protein extracted as described in the biochemical analysis section.

### Biochemical analysis

Brains and spinal cords were homogenized in lysis buffer (RIPA buffer (Pierce), 2% SDS, protease (Roche) and phosphatase (Thermo Fisher Scientific) inhibitors). Lysates were sonicated and microcentrifuged for 20 min at 13,000*g* at room temperature and soluble fractions collected. Proteins were separated on NuPAGE 4% to 12% bis-tris gels (Invitrogen) and transferred to nitrocellulose membranes (Bio-Rad Laboratories). Membranes were blocked in 5% milk in PBS-T (PBS, 0.1% Tween-20) for 1 h at room temperature. The membranes were incubated overnight at 4 °C with the following primary antibodies: C9orf72 (12E7, kindly donated by Prof. Dr. Manuela Neumann; 1:4 dilution), COL6 (ab182744, Abcam; 1:1,000), β-Actin (A2228, Sigma-Aldrich; 1:5,000 dilution), Phospho-TDP-43 (Ser409/410) (22309-1-AP, Proteintech; 1:1,000 dilution), Calnexin (sc-6465, Santa Cruz Biotechnology; 1:1,000 dilution). After three washes in PBS-T, membranes were incubated with secondary HRP-conjugated antibodies for 1 h at room temperature. After three washes in PBS-T, signals were visualized by chemiluminescence (Amersham imager 680) and quantifications performed using ImageJ software.

MSD immunoassays were performed as previously described^60,61^, using our custom rabbit anti-(GR)_7_ antibody^60,61^, and PR32B3 (Helmholtz Zentrum 2 µg ml^−1^) for capture and detection. GFP levels were measured by the GFP ELISA Kit (ab171581, Abcam) according to manufacturer’s instructions.

### qPCR with reverse transcription

Tissues were dissected and flash-frozen. Total RNA was extracted with miRNeasy Micro Kit (Qiagen) and reverse-transcribed using SuperScript IV Reverse Transcriptase (Invitrogen) with random hexamers and Oligo(dT)_20_ primers. Gene expression was determined by quantitative real-time PCR using a LightCycler and SYBR green (Roche). Relative gene expression was determined using the ΔΔCT (cycle threshold) method. Primers for mouse *C9orf72* are: 5′-TGAGCTTCTACCTCCCACTT-3′ and 5′-CTCTGTGCCTTCCAAGACAAT-3′. Primers to amplify the knock-in sequence are: 5′-GCGGCGAGTGGCTATTG-3′ (primer located within mouse *C9orf72* gene at exon boundary 1-2) and 5′-GGGTAAGCGTAATCTGGAACATC-3′ (sequence within the HA-tag sequence). Primers for mouse *Actin* are: 5′-CTGGCTCCTAGCACCATGAAGAT-3′ and 5′-GGTGGACAGTGAGGCCAGGAT-3′.

Total RNA from cells was extracted with ReliaPrep RNA Cell Miniprep System (Promega) and reverse-transcribed using SuperScript IV VILO Master Mix (Invitrogen). Gene expression was determined as described above. Primers for human *TGFB1* are: 5′-GGCTACCATGCCAACTTCT-3′ and 5′-CCGGGTTATGCTGGTTGT-3′. Primers for human *COL6A1* are: 5′-ACTTCGTCGTCAAGGTCATC-3′ and 5′-CATCTGGCTGTGGCTGTA-3′. Primers for human *GAPDH* are: 5′-ACTAGGCGCTCACTGTTCT-3′ and 5′-CCAATACGACCAAATCCGTTG-3′.

*Drosophila* were flash-frozen in liquid nitrogen. Total RNA was isolated using TRIzol reagent (Thermo Fisher Scientific). RNA samples were treated with TURBO DNase (Thermo Fisher Scientific), and converted to complementary DNA using oligod(T) primers and Superscript II reverse transcriptase (Invitrogen). Gene expression was determined by quantitative real-time PCR using QuantStudio 6 Flex Real-Time PCR System (Applied Biosystems). Relative gene expression was determined using the ΔΔCT method. For qPCR, primers for *Drosophila daw* are: 5′-GGATCAGCAGAAGGACTCCAA-3′ and 5′-CAGTGTTTGATGGGCCACTC-3′. Primers for *Drosophila Mp* are: 5′-CTGGGCACCTTCAAGGCATT-3′ and 5′-ATCGCCACGAGTGTTCACC-3′. Primers for *Drosophila Tubulin* are: 5′-TGGGCCCGTCTGGACCACAA-3′ and 5′-TCGCCGTCACCGGAGTCCAT-3′.

### Immunohistochemistry

Mice were perfused with prechilled PBS and then 4% paraformaldehyde (PFA). Brains and spinal cords were dissected and postfixed in 4% PFA at 4 °C for 2 h. After fixation, brains and spinal cords were washed with PBS, allowed to sink in 30% sucrose solution at 4 °C, then stored in 0.02% sodium azide at 4 °C until further processing. Brains and spinal cords were embedded in optimal cutting temperature (OCT) compound (Tissue Tek, Sakura), and 10-μm sections were cut with a cryostat (CM1860 UV, Leica Microsystem). For immunofluorescence, cryosections were washed three times in PBS and blocked in 5% BSA, 1% normal goat serum and 0.2% Triton-X in PBS for 1 h at room temperature. Sections were then incubated with primary antibodies in blocking solution overnight at 4 °C. After three washes with PBS, sections were incubated for 1 h at room temperature in blocking solution with secondary antibodies conjugated with Alexa 488, 546, 594 and 633 (Invitrogen). After three washes in PBS, sections were mounted with ProLong Gold Antifade Mountant with DAPI (Invitrogen).

Alternatively, after fixation, brains were washed with PBS, processed overnight using an automated tissue processor (Leica ASP300) and embedded in paraffin (Leica EG1150H). For immunofluorescence, 5-µm sections mounted on glass slides were incubated for 2 h at 60 °C. Sections were deparaffinized in xylene and rehydrated in decreasing grades of alcohol. Slides were incubated in methanol/hydrogen peroxide (0.3%) solution for 10 min at room temperature to block endogenous peroxidase activity. For antigen retrieval, slides were then transferred to a boiling solution of 0.1 M citrate buffer (pH 6.0) and pressure cooked at maximum pressure for 10 min. For immunofluorescence, slides were then blocked in 10% milk for 1 h at room temperature and incubated with primary and secondary antibodies as described above. For 3,3ʹ-diaminobenzidine (DAB) staining, slides were incubated in methanol/hydrogen peroxide (0.3%) solution for 10 min at room temperature and then blocked in 10% milk for 1 h at room temperature and incubated with primary antibody in PBS overnight at 4 °C. After three washes with PBS, sections were incubated for 30 min at room temperature in biotinylated secondary antibody (Vector Laboratories) in PBS. Slides were then washed in PBS and incubated in VECTASTAIN Elite ABC-HRP Kit, Peroxidase (Vector Laboratories) for 30 min at room temperature. Sections were washed three times with PBS and incubated in DAB chromogen (Abcam). Slides were then dehydrated in increasing grades of alcohol (70%, 95% and 100% ethanol), cleared in xylene and mounted with DPX mounting medium (Sigma-Aldrich).

The primary antibodies used were: HA clone 3F10 (11867423001, Roche; 1:100 dilution), NEUN (ABN91, Millipore; 1:500 dilution), IBA1 (019-19741, FUJIFILM Wako Pure Chemical; 1:500 dilution), GFAP (AB5804, Abcam; 1:500 dilution), GFAP (2.2B10, Invitrogen; 1:500 dilution), S100β (ab41548, Abcam; 1:300 dilution), CD68 (MCA1957, Bio-Rad Antibodies; 1:200 dilution), TDP-43 (12892-1-AP, Proteintech; 1:400 dilution), COL6 (ab182744, Abcam; 1:200), CTIP2 (ab18465, Abcam; 1:500), polyPR (PR32B3, Helmholtz Zentrum; 1:100).

Images were taken using a Zeiss LSM 880 confocal microscope or ZEISS Axio Scan.Z1 slide scanner. Image analyses were performed using ImageJ, Imaris or QuPath-0.3.2 software.

### Surgical procedures for in vivo recordings

Surgical and experimental procedures were conducted in accordance with the Animal (Scientific Procedures) Act 1986, approved by the Animal Welfare and Ethical Review Body at UCL and performed under an approved UK Home Office project license at UCL. Before surgical procedures, WT, PR(400) and GR(400) mice were anesthetized with isoflurane (3–4% induction, 1.5–2% for maintenance) and given subcutaneous carprofen for pain relief. The animal’s head was shaved to remove fur and the animal placed in a stereotaxic frame (WPI). Exposed skin was disinfected using diluted chlorhexidine and cleaned with ethanol. Anesthetic depth was confirmed by monitoring pedal reflex and breathing rate. Following a small incision, the skin overlying the skull was retracted and connective tissue over the skull cleared. A small craniotomy overlying motor cortex was then performed using a hand-held microdrill (WPI). In some animals, a silver chloride wire was then attached to a small indentation in the skull overlying cerebellum using cyanoacrylate glue (to act as a ground/reference electrode) and dental cement (Jet) was used to secure the wire and build a well encircling the craniotomy. Animals were then transferred directly to the electrophysiology station. In remaining animals, a glass coverslip (5-mm diameter) was placed over the craniotomy, and dental cement (Jet) applied to secure the cranial window and cover remaining exposed skull. On completion of these procedures, a subcutaneous injection of buprenorphine (0.1 mg kg^−1^) was administered for immediate postsurgical pain relief. After recovery, animals were returned to the holding room in single-housed conditions. Recordings were performed at least 2 weeks following recovery.

### In vivo two-photon Ca^2+^ imaging and analysis

In vivo two-photon Ca^2+^ imaging of motor cortex was performed under light isoflurane anesthesia (~1%) using a custom-built resonant-scanning two-photon microscope (Independent NeuroScience Services) controlled by ScanImage (MBF Bioscience), and equipped with a Coherent Chameleon Discovery NX tunable laser and a ×16, 0.8 numerical aperture, Nikon water immersion objective. Imaging of neuronal activity was performed at a wavelength of 1,070 nm and fluorescence detected with a GaAsP photomultiplier tube (Hamamatsu). Images (512 × 512 pixels) were acquired at 30-Hz frame rate, and each field-of-view was recorded for at least 5 min. Image analysis was performed with Suite2p (ref. ^62^) and custom MATLAB scripts. The recorded image stacks were loaded into Suite2p for motion correction, region of interest (ROI) detection and Ca^2+^ signal extraction. For each detected ROI (putative cell somata), the neuropil-corrected signal was extracted by subtracting the neuropil fluorescence signal surrounding the ROI (*F*_n_) from the raw fluorescence signal within the ROI (*F*): *F*_corr_(*t*) = *F*(*t*) − 0.7 × *F*_n_(*t*). The baseline fluorescence (*F*_0_) was estimated by using robust mean estimation and relative fluorescence change (Δ*F*/*F* = (*F*_corr_(*t*) − *F*_0_)/*F*_0_) over time (*t*) was generated for each ROI. Ca^2+^ transients were identified as relative changes in Δ*F*/*F* that were two times larger than the standard deviation of the noise band. Following automatic peak detection, the peaks were inspected using custom-written MATLAB scripts and manually curated to exclude false positives and include false negatives. Silent and hyperactive neurons were defined as those with individual activity rates of 0 and >3 transients per min, respectively.

### In vivo Neuropixels recordings and analysis

For recordings following surgical recovery, a small aperture overlying the motor cortex was made in the glass coverslip using a diamond-tipped drill bit and microdrill, and a silver chloride ground/reference electrode affixed to the edge of the cranial window. In all animals, a Neuropixels probe^63^ (IMEC) was connected to the ground/reference electrode and slowly implanted into motor cortex using stereotactic procedures at a rate ~5–10 µm s^−1^ under remote micromanipulator control (QUAD, Sutter Instruments) and visualized through a microscope (GT Vision). Following successful implantation, the cranial well was filled with warm sterile saline and the brain allowed to rest for at least 45 min before recordings. A 10-min recording of spontaneous neuronal activity was then performed in each animal under light isoflurane anesthesia (~1%). Neuropixels recordings (30-kHz sampling rate) were processed and automatically spike sorted using Kilosort3 using default parameters^64^. Processed data were then imported into PHY software (https://github.com/cortex-lab/phy) for interactive visualization of putative cortical clusters, and data manually inspected and curated to exclude false positives and include false negatives, and improve clustering through merging where appropriate. Following curation, spike-sorted data were analyzed using custom-written MATLAB scripts and subjected to an additional quality control where only units in which less than 1% of associated spikes violated the physiological refractory period of 2 ms were included for further analysis. Mean firing rates of all units (as log_10_Hz) across the entire 10-min recording session were calculated over 1-s time-bins for superficial cortex (0–400 µm below surface) and layer 5 (550–800 µm below surface). LFP data for the entire 10-min recording session were low pass filtered and decimated from 2.5 kHz to 500 Hz using an eighth order Chebyshev Type 1 IIR filter and common average referenced. LFP data were averaged across cortical channels and mean power in the slow (0.1–1 Hz), mid gamma (40–90 Hz) and high gamma (90–120 Hz) frequency bands estimated using Welch’s technique (40-s windows with 50% overlap, MATLAB function ‘pwelch’). Data were tested for normal distributions using Kolmogorov–Smirnov or Anderson–Darling tests.

### Locomotor, grip strength and body weight assessment

Behavioral tests were performed monthly from 3 to 9 months of age, and in 12-month-old mice. Motor coordination was measured by rotarod analysis (Ugo Basile). A power calculation using GPower predicted that for an effect size of 10% deviation from the group mean, with a power of 0.85 and an alpha of 0.05, groups sizes of 28 were needed, so we used 14 females and 14 males per group. Mice were trained the week before starting the test. Then, mice received a session which included three trials of accelerated rotarod for a maximum of 300 s. Trials started at 4-r.p.m. speed and accelerated up to 40 r.p.m. in 4 min; the final minute of the test was performed at 40 r.p.m. The average of the three trials was used. A grip strength meter (Bioseb) was used to measure forelimb and hindlimb grip strength. The highest muscle force score of three independent trials was used. Body weight was measured weekly from 3 months of age. Mice were randomized into different experimental groups and the operator was blind to genotype.

### Motor neuron counts

The 10-μm-thick OCT-embedded spinal cord sections were stained with Cresyl Violet and motor neurons located within the sciatic motor pool were counted in each ventral horn on 35 sections, collected every 60-μm of tissue, covering L3 to L5 levels of the spinal cord.

### In vivo isometric muscle tension physiology

Isometric muscle tension physiology was performed as previously described^65,66^. Briefly, under deep anesthesia (isoflurane inhalation via nose cone), hindlimbs were immobilized and the distal tendons of the tibialis anterior and EDL muscles of both hindlimbs were exposed and consecutively attached to force transducers in parallel. Sciatic nerves were exposed bilaterally, at mid-thigh level, severed and the distal stumps placed in contact with stimulating electrodes. EDL muscle MUNEs were determined by gradually increasing the amplitude of repeated square wave stimuli, thereby inducing stochastic changes in contractile force. The total number of motor units recruited over the full range of amplitudes was counted for individual muscles and averaged for each genotype.

### Mouse hindlimb lumbrical muscles preparation and NMJ imaging and analysis

Muscles were dissected and NMJs were stained as previously described^67^. NMJs were analyzed with the NMJ‐morph workflow. The following antibodies were used: mouse anti‐neurofilament (2H3, Developmental Studies Hybridoma Bank (DSHB), supernatant; 1:250 dilution), mouse pan anti‐synaptic vesicle 2 (SV2, DSHB, supernatant; 1:25 dilution) and Alexa Fluor 555 α‐bungarotoxin (α‐BTX; Life Technologies, B35451; 1:1,000 dilution).

### Proteomic analysis

Mouse lumbar spinal cords and cortices were solubilized in SDS lysis buffer (2% (wt/vol) SDS in 100 mM triethylammonium bicarbonate supplemented with Roche protease mini and Phos-STOP cocktail tablets). Automated homogenization was performed using the Precellys evolution homogenizer and Bioruptor-assisted sonication. Protein estimation was by BCA assay and protein quality was confirmed by SDS–PAGE. Lysate (200 µg) was aliquoted and processed using S-Trap-assisted On-column tryptic digestion as described previously (10.17504/protocols.io.bs3tngnn). Peptide eluates were then subjected to TMT labeling and high-pH fractionation for liquid chromatography with tandem mass spectrometry (LC–MS/MS); a total of 96 fractions were collected and pooled to 48 fractions, vacuum dried and stored at −80 °C until LC–MS/MS analysis.

LC–MS/MS analysis: a total of 48 basic reverse-phase liquid chromatography fractions were prepared for mass spectrometry analysis using an Orbitrap Tribrid Lumos mass spectrometer in-line with an Ultimate 3000 RSLC nano-liquid chromatography system. The mass spectrometer was operated in a data-dependent SPS-MS3 mode at a top speed for 2 s. Full scan was acquired at 120,000 resolution at 200 *m*/*z* measured using an Orbitrap mass analyzer in the scan range of 350–1,500 *m*/*z*. The data-dependent MS2 scans were isolated using a quadrupole mass filter with 0.7-Th isolation width and fragmented using normalized 32% higher-energy collisional dissociation and detected using an ion trap mass analyzer which was operated in a rapid mode.

Data analysis for mouse tissue: spinal cord raw mass spectrometry data were searched with MaxQuant software suite (v.2.0.1.0)^68^ against the Uniprot Mouse database appended with (GR)400 and (PR)400 sequences for C9orf72, and a common contaminant list exists within MaxQuant. FDR was set at 1% for both protein and peptide-spectrum match levels. The protein group output files were further processed using Perseus software suite (v.1.6.15.0)^69^ for downstream statistical analysis. Two-sided Welch’s *t*-test with 5% FDR multiple-correction was performed to identify differentially regulated proteins. GO analysis was performed on differentially regulated proteins using enrichR software^70^.

Reanalysis of C9orf72 iPS cell-derived motor neurons: we downloaded the SWATH mass spectrometry raw data from the CHORUS repository containing ten control and seven ALS samples. The .wiff and .wiff.scan files were then converted to mzML using mass spectrometry msConvert with the peak picking filter added^71^. Converted files were then searched using Spectronaut software suite version Rubin: 15.7.220308.50606 (ref. ^72^) using a direct-data-independent acquisition strategy. A FASTA file from the Human UniProt database was used to generate a predicted library within Spectronaut and a search was performed using the Pulsar search algorithm with default search parameters. The output protein group file was then processed using the Perseus software suite as described above to perform two-sided Welch’s *t*-test with 5% FDR multiple-correction to identify differentially regulated proteins between ALS and control samples.

### Microarray analysis

We analyzed the transcriptional signatures in laser-captured spinal motor neurons from postmortem C9ALS patients^32^. Raw microarray data are available from the Gene Expression Omnibus (GEO) with accession number GSE56504. We performed Gene Set Enrichment Analysis (GSEA) using the gseGO function from the clusterProfiler R package^73^. Differentially expressed genes were ranked and then subjected to GSEA. The top enriched gene sets included all had normalized enrichment score > 0.

### IPA

Data were analyzed with the use of QIAGEN IPA (QIAGEN, https://digitalinsights.qiagen.com/IPA). We used the user dataset as the reference dataset, and *P* = 0.05 and log fold change −1.5 to 1.5.

### Human frontal cortex RNA-seq data analysis

We used previously published RNA-seq data from the frontal cortex of pathologically diagnosed patients with frontotemporal lobar degeneration (with and without ALS) and control samples. We extracted the summary statistics and residual expression values for our gene(s) or pathways of interest from the differential gene expression and Weighted Gene Co-expression Network Analysis with adjustment for cell-type markers^33^.

### iPS cell differentiation and lentiviral transduction

The WTC11 iPS cell line harboring a doxycycline-inducible NGN2 cassette (kind gift of Dr. Michael E. Ward) was differentiated into cortical neurons (i^3^Neurons) as previously described^35^. On DIV 3, neural progenitor cells were dissociated with accutase and replated onto poly-l-ornithine (Merck) and laminin-coated plates in neuronal maintenance media: Neurobasal (Gibco), supplemented with 1 × B27 (Gibco), 10 ng ml^−1^ BDNF (PeproTech), 10 ng ml^−1^ NT-3 (PeproTech) and 1 µg ml^−1^ laminin. Neurons were plated at the desired ratio (typically 6 × 10^5^ cells per well of a 6-well plate). At DIV 3, lentivirus was added to i^3^Neurons to overexpress (GR)_50_ or GFP constructs under the control of the neuron-specific human synapsin 1 promoter as previously described^36^. From DIV 3 to DIV 7, cells were maintained in neuronal maintenance media and imaged on the Incucyte S3 Live-Cell Analysis System using a confluency mask to quantify and track cell survival.

### *Drosophila* stocks and maintenance

*Drosophila* stocks were maintained on SYA food (15 g l^−1^ agar, 50 g l^−1^ sugar, 100 g l^−1^ brewer’s yeast, 30 ml l^−1^ nipagin (10% in ethanol) and 3 ml l^−1^ propionic acid) at 25 °C in a 12-h light/dark cycle with 60% humidity. The UAS-(GR)36 flies have been previously described^7^. The elav-GS stock was obtained from a kind gift of H. Tricoire^74^. The fly lines GMR-Gal4 (Bloomington no. 9146), UAS-daw RNAi (Bloomington no. 50911 and no. 34974) and UAS-Mp RNAi (Bloomington no. 52981 and no. 28299) were obtained from the Bloomington Drosophila Stock Center.

### *Drosophila* ortholog prediction

The Drosophila RNAi Screening Center Integrative Ortholog Prediction Tool (DIOPT; http://www.flyrnai.org/diopt) was used to search for orthologues of TGF-β1, COL6A1, COL6A2 and COL6A3. DIOPT predicted *daw* as the *Drosophila* orthologue of *TGF-β1*, and *Mp* as the *Drosophila* orthologue of human COL6A1, COL6A2 and COL6A3.

### *Drosophila* eye phenotype analysis

Flies carrying the UAS-daw RNAi or UAS-Mp RNAi construct were crossed to the GMR-GAL4, UAS-(GR)36 line at 25 °C. Two-day-old adult F1 flies were imaged using a stereomicroscope, with female eyes used for analyses. All eye images were obtained under the same magnification; eye area was calculated from each image using ImageJ (ref. ^75^).

For toxicity scoring, 2-day-old adult F1 flies were examined with a dissecting microscope. Adult flies were separated into four groups based on the severity of the rough eye phenotype: WT, low, medium and high. The percentage of flies in each category was calculated. The results were analyzed by chi-squared test.

### *Drosophila* lifespan assays

Lifespan assays were carried out as previously described^76^. The parental generation of the genotype used in each lifespan assay was allowed to lay for 24 h on grape agar plates supplemented with yeast. Eggs were placed at a standard density into bottles containing SYA medium. Adult experimental flies were allowed to emerge and mate for 48 h before being lightly anesthetized with CO_2_, and females randomly allocated onto SYA containing RU486 (200 μM) or ethanol vehicle at a standard density per vial (*n* = 15). Flies were transferred to fresh vials three times per week, with deaths scored at least three times per week. Escaping flies were censored from the data. Log-rank test was performed using Microsoft Excel (template available at http://piperlab.org/resources/).

### iPS cell-derived neuronal differentiation and glutamate toxicity

Non-neurological control and *C9orf72* iPS cells were obtained from the Answer ALS repository at Cedars Sinai (see Supplementary Table 1 for demographics) and maintained in mTeSR Plus medium at 37 °C with 5% CO_2_. iPSNs were differentiated in accordance with the diMNs protocol previously described^28,38,77^ and maintained at 37 °C with 5% CO_2_. On day 32 of differentiation, iPSNs were dissociated with accutase to facilitate the generation of a single-cell suspension. A total of 5 × 10^6^ iPSNs were nucleofected with 4 µg of plasmid DNA (Origene) in suspension as previously described^28,38^. Following nucleofection, 100 µl of cell suspension was plated in each well (total of six wells per cuvette) of a glass-bottom or plastic 24-well plate for propidium iodide (PI) and Alamar Blue toxicity and viability experiments, respectively. Medium was exchanged daily for a total of 7 d to facilitate the removal of iPSNs that failed to recover postnucleofection. On the day of the experiment (day 39 of differentiation), iPSN medium was replaced with artificial cerebrospinal fluid solution containing 10 µM glutamate. For those iPSNs undergoing Alamar Blue viability assays (plastic dishes), Alamar Blue reagent was additionally added to each well according to the manufacturer’s protocol at this time. Following incubation, iPSNs for PI cell death assays were incubated with PI and NucBlue live ready probes for 30 min and subjected to confocal imaging as previously described^28^. The numbers of PI spots and nuclei were automatically counted in FIJI. Alamar Blue cell viability plates were processed according to the manufacturer’s protocol. As a positive control, 10% Triton-X-100 was added to respective wells 1 h before processing.

### Statistical analysis

All data are presented as mean ± s.e.m. Statistical differences of continuous data from two experimental groups were calculated using unpaired two-sample Student’s *t*-test. Comparisons of data from more than two groups were performed using a one-way analysis of variance (ANOVA), followed by Bonferroni correction for multiple comparisons, or Tukey’s multiple comparisons, or Fisher’s least significant difference procedure. When two independent variables were available, comparisons of data from more than two groups were performed using a two-way ANOVA followed by Bonferroni correction for multiple comparisons, or Tukey’s multiple comparisons. Data distribution was tested for normality using the Kolmogorov–Smirnov test; when normality could not be tested, we assumed data distribution to be normal. The statistical significance threshold was set at *P* < 0.05, unless otherwise indicated. Statistical methods were used to predetermine sample sizes.

### Data collection

Data collection and analysis were performed blind to the conditions of the experiments.

### Reporting summary

Further information on research design is available in the Nature Portfolio Reporting Summary linked to this article.

## Online content

Any methods, additional references, Nature Portfolio reporting summaries, source data, extended data, supplementary information, acknowledgements, peer review information; details of author contributions and competing interests; and statements of data and code availability are available at 10.1038/s41593-024-01589-4.

## Supplementary information

Supplementary Information Supplementary Table 1.

Reporting Summary

## Source data

Source Data Fig. 1 Statistical source data.

Source Data Fig. 1 Uncropped western blots.

Source Data Fig. 3 Statistical source data.

Source Data Fig. 4 Statistical source data.

Source Data Fig. 5 Statistical source data.

Source Data Fig. 5 Unprocessed western blots.

Source Data Fig. 6 Statistical source data.

Source Data Fig. 7 Statistical source data.

Extended Data Fig. 2 Statistical source data.

Extended Data Fig. 3 Statistical source data.

Extended Data Fig. 4 Statistical source data.

Extended Data Fig. 5 Statistical source data.

Extended Data Fig. 6 Statistical source data.

Extended Data Fig. 7 Statistical source data.

Extended Data Fig. 8 Statistical source data.

Extended Data Fig. 8 Unprocessed western blots.

Extended Data Fig. 10 Statistical source data.

## Data Availability

The datasets generated and analyzed during the present study are available from the corresponding authors upon request. Proteomics data from cortex and spinal cord, MS raw data and search output files have been deposited to the PRIDE ProteomeXchange Consortium via the PRIDE partner repository with the dataset identifier PXD047502. Source data are provided with this paper.

## Code availability

The customized codes present in the study are available from the corresponding authors upon request.
