# Supplementary material for: PolyGR and polyPR knock-in mice reveal a conserved neuroprotective extracellular matrix signature in C9orf72 ALS/FTD neurons
Source: Nat Neurosci. 2024 Feb 29;27(4):643–55. doi: 10.1038/s41593-024-01589-4 (PMC11001582; doi:10.1038/s41593-024-01589-4)
Supplement: Supplementary file 1 — Supplementary Table 1. [file 41593_2024_1589_MOESM1_ESM.pdf]

nature neuroscience

Article https://doi.org/10.1038/s41593-024-01589-4

PolyGR and polyPR knock-in mice reveal a conserved neuroprotective extracellular matrix signature in *C9orf72* ALS/FTD neurons

In the format provided by the authors and unedited

iPSC Line Name	Source	Clinical Diagnosis	Age at Time of Collection	Sex
CS0201	Cedars-Sinai	Non-neurologic control	56	Female
CS0002	Cedars-Sinai	Non-neurologic control	51	Male
CS0206	Cedars-Sinai	Non-neurologic control	72	Female
CS9XH7	Cedars-Sinai	Non-neurologic control	53	Male
CS8PAA	Cedars-Sinai	Non-neurologic control	58	Female
CS1ATZ	Cedars-Sinai	Non-neurologic control	60	Male
CS0NKC	Cedars-Sinai	C9orf72	52	Female
CS0LPK	Cedars-Sinai	C9orf72	67	Male
CS0BUU	Cedars-Sinai	C9orf72	63	Female
CS7VCZ	Cedars-Sinai	C9orf72	64	Male
CS6ZLD	Cedars-Sinai	C9orf72		Female
CS8KT3	Cedars-Sinai	C9orf72	60	Male

Origin
PBMC
PBMC
PBMC
PBMC
PBMC
PBMC
PBMC
PBMC
PBMC
PBMC
PBMC
PBMC
