# Supplementary material for: PolyGR and polyPR knock-in mice reveal a conserved neuroprotective extracellular matrix signature in C9orf72 ALS/FTD neurons
Source: Nat Neurosci. 2024 Feb 29;27(4):643–55. doi: 10.1038/s41593-024-01589-4 (PMC11001582; doi:10.1038/s41593-024-01589-4)
Supplement: Supplementary file 2 — Reporting Summary [file 41593_2024_1589_MOESM2_ESM.pdf]

nature portfolio

Corresponding author(s): Adrian Isaacs and Elizabeth Fisher

Last updated by author(s): Dec 18, 2023

Reporting Summary

Nature Portfolio wishes to improve the reproducibility of the work that we publish. This form provides structure for consistency and transparency in reporting. For further information on Nature Portfolio policies, see our Editorial Policies and the Editorial Policy Checklist.

Statistics

For all statistical analyses, confirm that the following items are present in the figure legend, table legend, main text, or Methods section.

n/a	Confirmed	
☐	☒	The exact sample size (*n*) for each experimental group/condition, given as a discrete number and unit of measurement
☐	☒	A statement on whether measurements were taken from distinct samples or whether the same sample was measured repeatedly
☐	☒	The statistical test(s) used AND whether they are one- or two-sided<br>*Only common tests should be described solely by name; describe more complex techniques in the Methods section.*
☒	☐	A description of all covariates tested
☐	☒	A description of any assumptions or corrections, such as tests of normality and adjustment for multiple comparisons
☐	☒	A full description of the statistical parameters including central tendency (e.g. means) or other basic estimates (e.g. regression coefficient) AND variation (e.g. standard deviation) or associated estimates of uncertainty (e.g. confidence intervals)
☐	☒	For null hypothesis testing, the test statistic (e.g. *F*, *t*, *r*) with confidence intervals, effect sizes, degrees of freedom and *P* value noted<br>*Give P values as exact values whenever suitable.*
☒	☐	For Bayesian analysis, information on the choice of priors and Markov chain Monte Carlo settings
☒	☐	For hierarchical and complex designs, identification of the appropriate level for tests and full reporting of outcomes
☒	☐	Estimates of effect sizes (e.g. Cohen's *d*, Pearson's *r*), indicating how they were calculated

Our web collection on statistics for biologists contains articles on many of the points above.

Software and code

Policy information about availability of computer code

Data collection	No custom code used, just standard analysis software.
Data analysis	GraphPad Prism version 9, ImageJ 1.54f, QuPath-0.3.2, Imaris Cell Imaging Software 9.8 (Bitplane).

For manuscripts utilizing custom algorithms or software that are central to the research but not yet described in published literature, software must be made available to editors and reviewers. We strongly encourage code deposition in a community repository (e.g. GitHub). See the Nature Portfolio guidelines for submitting code & software for further information.

Data

Policy information about availability of data

All manuscripts must include a data availability statement. This statement should provide the following information, where applicable:

- Accession codes, unique identifiers, or web links for publicly available datasets
- A description of any restrictions on data availability
- For clinical datasets or third party data, please ensure that the statement adheres to our policy

Proteomics data is provided in excel format (see source data file). Raw data available in PRIDE, accession number PXD047502.

Human research participants

Policy information about studies involving human research participants and Sex and Gender in Research.

Reporting on sex and gender	NA
Population characteristics	NA
Recruitment	NA
Ethics oversight	NA

Note that full information on the approval of the study protocol must also be provided in the manuscript.

Field-specific reporting

Please select the one below that is the best fit for your research. If you are not sure, read the appropriate sections before making your selection.

☒ Life sciences ☐ Behavioural & social sciences ☐ Ecological, evolutionary & environmental sciences

For a reference copy of the document with all sections, see nature.com/documents/nr-reporting-summary-flat.pdf

Life sciences study design

All studies must disclose on these points even when the disclosure is negative.

Sample size	For grip strength and rotarod analysis used the NC3Rs Experimental Design Assistant (EDA). EDA recommended a two-way ANOVA and we used GPower to perform our power calculation. For an effect size of 10% deviation from the group mean, with a power of 0.85 and an alpha of 0.05, groups sizes of 28 were specified. Sample size calculations were not performed for other experiments, numbers were based on those previously used in the literature.
Data exclusions	No data excluded
Replication	Experiments were performed on multiple mice per genotype with consistent results across mice.
Randomization	Mice were randomly assigned into experimental groups. Randomization not relevant for other experiments.
Blinding	Operator was blind to genotype

Reporting for specific materials, systems and methods

We require information from authors about some types of materials, experimental systems and methods used in many studies. Here, indicate whether each material, system or method listed is relevant to your study. If you are not sure if a list item applies to your research, read the appropriate section before selecting a response.

Materials & experimental systems

n/a	Involved in the study	
☐	☒	Antibodies
☐	☒	Eukaryotic cell lines
☒	☐	Palaeontology and archaeology
☐	☒	Animals and other organisms
☒	☐	Clinical data
☒	☐	Dual use research of concern

Methods

n/a	Involved in the study	
☒	☐	ChIP-seq
☒	☐	Flow cytometry
☒	☐	MRI-based neuroimaging

Antibodies

Antibodies used	Western blots: C9orf72 (12E7, kindly donated by Prof. Dr. Manuela Neumann; 1:4 dilution), COL6 (ab182744, Abcam; 1:1000), β-Actin (A2228, Sigma-Aldrich;1:5000 dilution), Phospho-TDP43 (Ser409/410) (22309-1-AP, Proteintech; 1:1000 dilution), Calnexin (sc-6465, Santa Cruz Biotechnology; 1:1000 dilution).<br>IHC: HA clone 3F10 (11867423001, Roche; 1:100 dilution), NEUN (ABN91, Millipore; 1:500 dilution), IBA1 (019-19741, FUJIFILM Wako Pure Chemical Corporation; 1:500 dilution), GFAP (AB5804, Abcam; 1:500 dilution), GFAP (2.2B10, Invitrogen; 1:500 dilution), S100β

	(ab41548, Abcam; 1:300 dilution), CD68 (MCA1957, Bio-Rad Antibodies; 1:200 dilution), TDP-43 (12892-1-AP, Proteintech; 1:400 dilution), COL6 (ab182744, Abcam; 1:200), CTIP2 (ab18465, Abcam; 1:500), mouse anti-neurofilament (2H3, Developmental Studies Hybridoma Bank (DSHB), Iowa City, IA, USA, supernatant; 1:250 dilution), mouse pan anti-synaptic vesicle 2 (SV2, DSHB, supernatant; 1:25 dilution), Alexa Fluor 555 α-bungarotoxin (α-BTX; Life Technologies, B35451; 1:1000 dilution), polyPR (PR32B3, Helmholtz Zentrum; 1:100). Secondary antibodies: HRP-conjugated anti-rat (P0450 Dako), HRP-conjugated anti-mouse (P0260 Dako), HRP-conjugated anti-rabbit (AQ132P Merck), Alexa Fluor 488 conjugated anti-rabbit (A11008, Invitrogen), Alexa Fluor 488 conjugated anti-mouse (A11001, Invitrogen), Alexa Fluor 546 conjugated anti-rabbit (A11035, Invitrogen), Alexa Fluor 546 conjugated anti-mouse (A11030, Invitrogen), Alexa Fluor 594 conjugated anti-rat (A11007, Invitrogen), Alexa Fluor 633 conjugated anti-chicken (A21103, Invitrogen).
Validation	C9orf72 antibody validated extensively by the generators see PMID: 30075745. COL6 - knockout validated by manufacturer. Phospho-TDP-43 - validated in >80 published papers. All other antibodies are standard cellular markers used in numerous previous studies.

Eukaryotic cell lines

Policy information about cell lines and Sex and Gender in Research

Cell line source(s)	WTC11 human iPSC line used, male, kindly provided by Dr Michael Ward.<br>Human iPSC lines (CS0201, CS0002, CS0206, CS9XH7, CS8PAA, CS1ATZ, CS0NKC, CS0LPK, CS0BUU, CS7VCZ, CS6ZLD, CS8KT3) from Cedars-Sinai used by Dr Alyssa N Coyne.
Authentication	None
Mycoplasma contamination	We confirmed the line was mycoplasma negative over the course of the experiments as we test regularly (monthly)
Commonly misidentified lines (See ICLAC register)	No commonly misidentified cell lines used.

Animals and other research organisms

Policy information about studies involving animals; ARRIVE guidelines recommended for reporting animal research, and Sex and Gender in Research

Laboratory animals	C57BL6/N backcrossed for at least 5 generation to C57BL6/J before use in the study, and analyzed from 3-19 months of age. Homozygous TAR4/4 mice overexpressing wild-type human TARDBP (TDP-43) at 24 days of age.
Wild animals	No wild animals were used in the study.
Reporting on sex	Males were used for all experiments in the main text, except in vivo two-photon calcium imaging and Neuropixels recording where females were used. Phenotyping was also performed on female mice, with similar results to males, and these data are included in the supplement data section.
Field-collected samples	No field collected samples were used in this study.
Ethics oversight	All procedures involving mice were conducted in accordance with the Animal (Scientific procedures) Act 1986 and the ARRIVE guidelines and were performed at University College London under an approved UK Home Office project licence reviewed by the Institute of Prion Diseases Animal Welfare and Ethical Review Body.

Note that full information on the approval of the study protocol must also be provided in the manuscript.
