# Supplementary figures and images for: PolyGR and polyPR knock-in mice reveal a conserved neuroprotective extracellular matrix signature in C9orf72 ALS/FTD neurons
Source: Nat Neurosci. 2024 Feb 29;27(4):643–55. doi: 10.1038/s41593-024-01589-4 (PMC11001582; doi:10.1038/s41593-024-01589-4)

A      Uncropped WB use for Fig 1f Brain

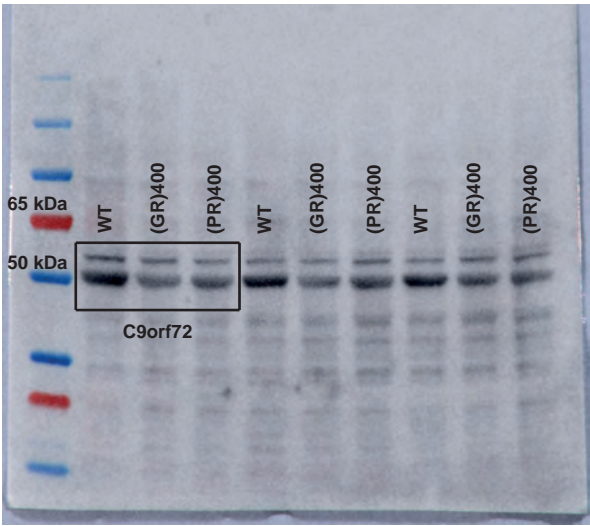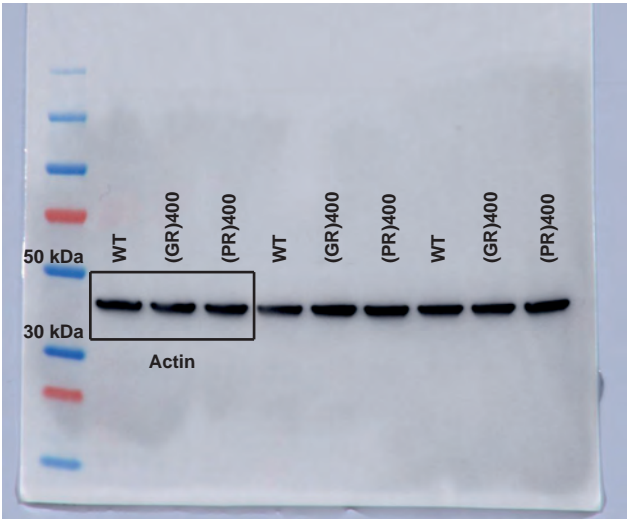

B      Uncropped WB use for Fig 1f Spinal cord

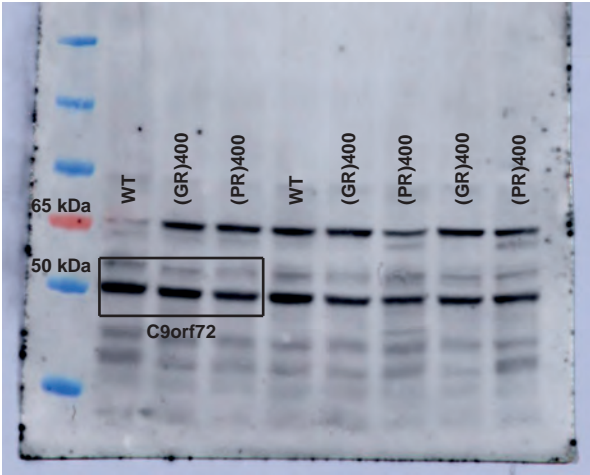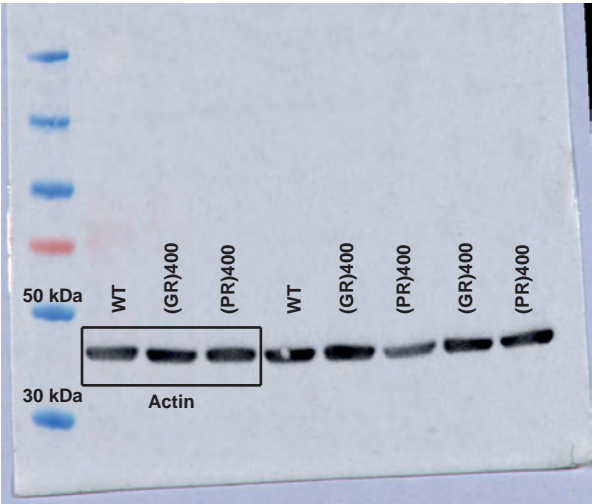

Supplement: Supplementary file 4 — Uncropped western blots. [file 41593_2024_1589_MOESM4_ESM.pdf]

A                      Uncropped WB use for Fig 5c Lumbar spinal cord

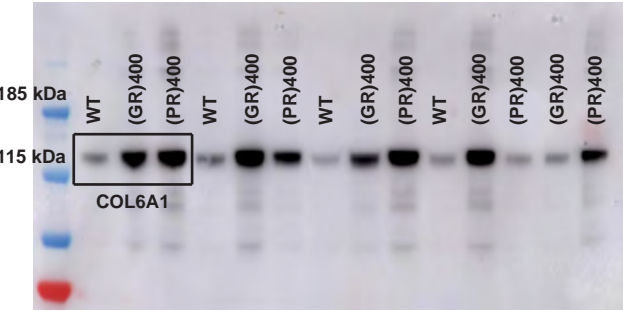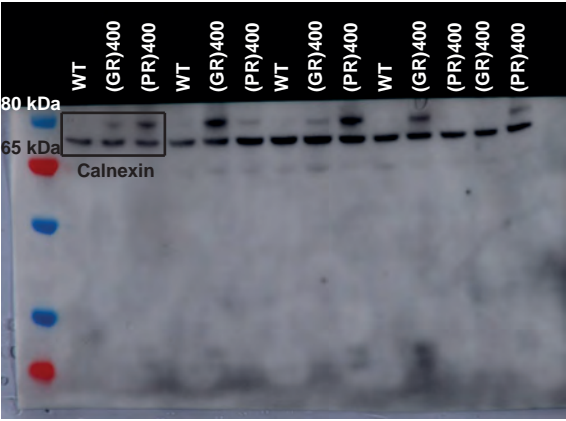

Supplement: Supplementary file 8 — Unprocessed western blots. [file 41593_2024_1589_MOESM8_ESM.pdf]

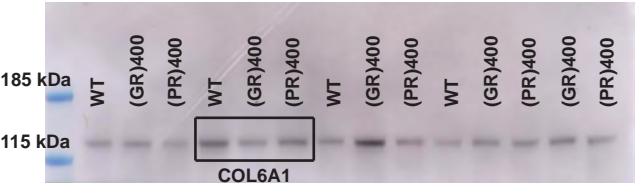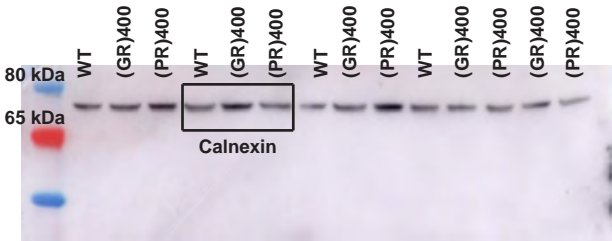

Supplement: Supplementary file 18 — Unprocessed western blots. [file 41593_2024_1589_MOESM18_ESM.pdf]
